# NKG2A-mediated immune modulation of natural killer cells by *Staphylococcus aureus*

**DOI:** 10.1093/jimmun/vkaf174

**Published:** 2025-08-07

**Authors:** Kate Davies, Al-Motaz Rizek, Sarah Edkins, Simon Kollnberger, Eddie C Y Wang, Matthias Eberl, Jonathan Underwood, James E McLaren

**Affiliations:** Division of Infection and Immunity, Cardiff University School of Medicine, Cardiff, United Kingdom; Division of Infection and Immunity, Cardiff University School of Medicine, Cardiff, United Kingdom; Division of Infection and Immunity, Cardiff University School of Medicine, Cardiff, United Kingdom; Systems Immunity University Research Institute, Cardiff University School of Medicine, Cardiff, United Kingdom; Division of Infection and Immunity, Cardiff University School of Medicine, Cardiff, United Kingdom; Systems Immunity University Research Institute, Cardiff University School of Medicine, Cardiff, United Kingdom; Division of Infection and Immunity, Cardiff University School of Medicine, Cardiff, United Kingdom; Systems Immunity University Research Institute, Cardiff University School of Medicine, Cardiff, United Kingdom; Division of Infection and Immunity, Cardiff University School of Medicine, Cardiff, United Kingdom; Systems Immunity University Research Institute, Cardiff University School of Medicine, Cardiff, United Kingdom; Division of Infection and Immunity, Cardiff University School of Medicine, Cardiff, United Kingdom; Systems Immunity University Research Institute, Cardiff University School of Medicine, Cardiff, United Kingdom; Department of Infectious Diseases, Cardiff and Vale University Health Board, Cardiff, United Kingdom; Division of Infection and Immunity, Cardiff University School of Medicine, Cardiff, United Kingdom; Systems Immunity University Research Institute, Cardiff University School of Medicine, Cardiff, United Kingdom

**Keywords:** bacteremia, natural killer cells, NKG2A, *Staphylococcus aureus*, superantigen

## Abstract

Natural killer (NK) cells are specialized lymphocytes that help protect against viruses and cancer. However, in the context of bacterial infections, NK cells can be harmful, rather than protective. Such immune pathogenesis by NK cells has been linked to the overproduction of proinflammatory cytokines like interferon-gamma (IFN-γ). In this context, IFN-γ–deficient mice display increased survival rates in response to *Staphylococcus aureus (S. aureus*) infection. However, little is known about how NK cells respond to *S. aureus* in humans, which causes life-threatening, invasive systemic infections with high mortality rates. In this study, we found that the peripheral blood of patients with bloodstream *S. aureus* infection was enriched for CD57^−^ NKG2A^+^ NK cells with greater cytokine-producing capacity, compared to healthy controls and those hospitalized with *Escherichia coli* bloodstream infections. As a possible mechanistic cause, superantigens from *S. aureus* promoted the expansion of CD57^−^ NKG2A^+^ NK cells that produced IFN-γ through a mechanism that appears to be IL-12 independent and exhibited reduced levels of CD16 compared to unstimulated NK cells. These data suggest that *S. aureus* bloodstream infection in humans promotes a phenotypic shift toward CD57^−^ NKG2A^+^ NK cells with greater IFN-γ–producing capacity, providing a plausible way to promote inflammation-driven disease pathogenesis.

## Introduction

*Staphylococcus aureus* (*S. aureus*) is an opportunistic, Gram-positive bacterium that commonly exists as a commensal organism in humans, asymptomatically colonizing specific anatomical sites, such as the nasal mucosa,^1^ skin, and gastrointestinal tract.^2^ Such residency requires the bacterium to overcome inbuilt host defense mechanisms, notably those orchestrated by innate (e.g. neutrophils) and adaptive (e.g. T cells) immune cells, which provide major forms of protective immunity.^3^ This commensalism is a risk factor for the human host to develop life-threatening, invasive infections such as bloodstream infections (or “bacteremia”) and endocarditis,^4^ which are associated with high mortality rates (30-day mortality at 20% to 30%).^5^^,^^6^  *S. aureus* is one of the most frequent causes of community- or hospital-acquired bacteremia, second only to the Gram-negative bacterium *Escherichia coli* (*E. coli*).^7^  *S. aureus* is also a leading cause of sepsis,^8^ a life-threatening condition caused by an exaggerated host immune response to infection that is estimated to underlie 1 in 5 deaths worldwide.^9^ Morbidity and mortality are exacerbated by its ability to establish deep-seated, metastatic infections despite seemingly effective antimicrobial therapy.^10^

The inherent threat from *S. aureus* bacteremia highlights the need to understand the pathophysiological interactions between this bacterium and the human immune system, including the immune evasive strategies that promote its persistence and survival. In this regard, *S. aureus* utilizes a complex arsenal of virulence factors and immune evasion proteins that prevent immunological elimination by neutrophils, macrophages, T cells, and antibodies.^11^ These include pore-forming toxins, such as leukocidin ED, Panton-Valentine leukocidin, and α-hemolysin, which bind to and directly kill neutrophils, macrophages, and/or T cells, and highly potent “superantigen” (SAg) exotoxins, which act to counteract and weaken host T cell defense mechanisms.^12–14^  *S. aureus* is capable of producing 26 distinct SAgs encoded with mobile genetic elements including toxic shock syndrome toxin 1 (TSST-1) and staphylococcal enterotoxins (SEs), such as SEA, SEB, and SEC1-3.^15^^,^^16^ Distinct *S. aureus* strains can encode multiple SAgs, although rarely produce >20 different SAgs at the same time.^15^^,^^17^ SAgs primarily function by cross-linking T cell receptors on αβ T cells to MHC class II molecules on the surface of antigen-presenting cells (APCs).^11^^,^^14^^,^^16^ Such engagement causes SAgs to overstimulate T cells, leading to excessive cytokine release that may contribute to the propagation of a cytokine “storm” while simultaneously driving the upregulation of co-inhibitory receptors on T cells that renders them hyporesponsive.^14^^,^^18–21^ Consequently, SAgs can drive life-threatening complications of *S. aureus* infection, such as toxic shock syndrome, necrotizing pneumonia, and infective endocarditis.^15^^,^^22^ SAgs can also bind costimulatory molecules,^23–26^ cytokine receptors,^27^ and extracellular matrix proteins,^28^ providing additional mechanisms of action for immune evasion. SAgs have therefore become targets of therapeutic intervention^29^^,^^30^ in an era of increasing antimicrobial resistance (e.g. methicillin-resistant *S. aureus* [MRSA]^31^) and the absence of a licensed vaccine against *S. aureus*.

Natural killer (NK) cells are innate immune cells of lymphocyte origin that play a specialist role in protecting the host against infectious pathogens and malignancy.^32^ Unlike T cells and B cells, NK cells lack somatically rearranged antigen receptors to mediate their function. Instead, NK cells utilize an array of germline-encoded activating and inhibitory receptors to recognize cell surface molecules of self or foreign origin, such as the killer cell immunoglobulin-like receptors (KIRs), natural cytotoxicity receptors (e.g. NKp30, NKp46), and the C-type lectin NKG2 receptors (e.g. NKG2A, NKG2C, NKG2D).^33^ Human NK cells typically emerge into the circulation from secondary lymphoid organs as CD3^−^ CD56^hi^ CD16^−^ cells^34^ expressing high levels of NKp30, NKG2A, and receptors for proinflammatory cytokines (e.g. IL-12, IL-18).^35^ These CD56^hi^ NK cells possess superior cytokine-producing capacity, despite having limited cytotoxic potential, but can differentiate into CD3^−^ CD56^dim^ CD16^+^ subsets that predominate in the circulation and undergo phenotypic changes to specialize their function toward receptor-driven cytotoxicity.^32^^,^^36^ These include expression of KIRs, CD57, and the activating NKG2C receptor while simultaneously losing NKG2A/CD94 expression.^36–38^ These highly differentiated NK cells can home to infected or dysregulated tissue sites to mediate their function, while many human organs retain populations of tissue-resident NK cells expressing established markers of residency, such as CD49a and CD103.^39–41^ NK cell responses to acute or resolving viral infections are typically cytokine-driven, with less differentiated NK cells predominantly responding to IL-12, IL-15, IL-18, and type I interferons (IFNs) produced during infection that promote their survival, proliferation, and effector function (e.g. through IFN-γ production).^35^^,^^42^^,^^43^ However, in response to chronic infections, such as human cytomegalovirus (HCMV), more differentiated NK cells expand and develop adaptive features, akin to T cells, such as clonal expansion and the formation of immunological memory.^44^^,^^45^ These adaptive NK cell expansions typically express NKG2C and CD57.^46^^,^^47^ NK cells are essential for antiviral protection since patients with inborn NK cell deficiencies are highly prone to severe or recurrent herpesvirus infections.^48^

In contrast to our knowledge of NK cell–mediated immunity to viruses, little is known about how NK cells respond to bacterial infections, especially in humans. NK cells can be activated by bacteria, directly through Toll-like receptors^49^ or indirectly by proinflammatory cytokines produced by bacteria-sensing APCs (e.g. dendritic cells).^50^^,^^51^ NK cell–deficient mice have increased bacterial burden and poor immune control in response to *Legionella pneumophila*^52^ and *Mycobacterium tuberculosis.*^53^ Furthermore, NK cell–produced IFN-γ helps to promote bacterial elimination during various infections, including *Klebsiella pneumoniae*,^52–54^ while memory-like NK cells mediate protective immune responses to *Streptococcus pneumoniae*^55^ and *Ehrlichia muris.*^56^ In contrast, NK cell–deficient or depleted mice have improved survival rates after *E. coli* or *Streptococcus pyogenes* infection, which has been linked to reduced levels of inflammation and lower production of proinflammatory cytokines, including IL-12 and IFN-γ.^57^^,^^58^ Furthermore, NK cells are thought to have a detrimental role in sepsis, including in immunocompromised hosts.^59^^,^^60^ This points to important pathogen-specific differences in the control and immunopathology of bacterial infections by NK cells. In the context of *S. aureus* infection, NK cells are in fact the major source of IFN-γ,^61^^,^^62^ and IFN-γ^−/−^ mice or those treated with depleting anti IFN-γ antibodies are more resistant to lethal *S. aureus* challenge.^63^^,^^64^ The fact that SAgs^65^^,^^66^ and other virulence factors such as the pore-forming toxins leukocidin ED,^67^ α-hemolysin,^68^ and β-hemolysin^69^ alter the functionality of NK cells, including increasing their production of IFN-γ, suggests that NK cell–released IFN-γ could be a driver of immune pathogenesis during *S. aureus* infection. However, studies of NK cell responses to systemic *S. aureus* infections have been confined to animal models.

To address this knowledge gap, we here examined the NK cell response to *S. aureus* or *E. coli* bacteremia in humans. We found that more polyfunctional CD56^dim^ NK cells with greater cytokine-producing potential were observed in patients with *S. aureus* bacteremia, compared to those hospitalized with *E. coli* bacteremia. These highly responsive NK cells were enriched for CD57^−^ NKG2A^+^ subsets. In support of a possible underlying mechanism, SAgs, namely TSST-1, promoted the *in vitro* expansion of CD57^−^ NKG2A^+^ NK cells that had reduced levels of CD16 and were capable of producing IFN-γ through an IL-12–independent mechanism. These data suggest that systemic *S. aureus* infection in humans promotes a phenotypic and functional shift toward IFN-γ–producing CD57^−^ NKG2A^+^ NK cells, providing a plausible way to promote inflammation-driven disease pathogenesis.

## Materials and methods

### Subjects and ethical approval

The recruitment of adult patients admitted to the University Hospital of Wales (UHW) with invasive bloodstream *S. aureus* or *E. coli* infections (bacteremia) was approved by the National Health Service Research Ethics Committee (REC) (REC reference 21/WA/0202, IRAS project ID 277598) and was sponsored by Cardiff University and the Cardiff and Vale University Health Board. Patients were excluded if they were pregnant, breastfeeding, or females of childbearing age in whom a pregnancy test had not been performed. Furthermore, participants considered too unwell, those with mental incapacity or language barriers that precluded adequate understanding, or those with reduced kidney function, indicated by an estimated glomerular filtration rate (eGFR) <45 ml/min/1.73 m^2^, were excluded. Healthy control blood donors (*n *= 12; age range 33–73 yrs [median 65 yrs]) were recruited as controls for the study if there was no history of recent (<1 yr) bloodstream *S. aureus* or *E. coli* infections; were over the age of 18; and had the capacity to consent or, in the event of temporarily reduced capacity, consent from the next of kin (or another suitable consultee) was obtained. Peripheral venous blood samples were collected from healthy controls at the point of enrollment. Additional adult patients admitted to the UHW with invasive bloodstream *S. aureus* or *E. coli* infections (bacteremia) were recruited through a separate study commenced through the Cardiff University Biobank (REC reference 23/WA/0073, IRAS project ID 321237). Here, patients with reduced kidney function (eGFR <45 ml/min/1.73 m^2^) were included. From both studies, peripheral venous blood samples were collected from patients with bacteremia at hospital admission (within 2 to 6 days post-diagnosis) from patients with *S. aureus* (*n *= 12; median 3 days postdiagnosis) or *E. coli* (*n *= 12; median 3 days post-diagnosis) bacteremia. Both study cohorts collectively comprised patients with microbiologically confirmed *S. aureus* (*n *= 12; age ranging from 27 to 93 yrs [median 63 yrs]) or *E. coli* (*n *= 12; age ranging from 31 to 74 yrs [median 67 yrs]) bacteremia. For experimental assays not involving bacteremia patients and study controls (i.e. experiments involving *in vitro* SAg stimulation assays), peripheral venous blood samples were collected from healthy volunteers and buffy coats purchased from the Welsh Blood Service. The use of venous blood samples from healthy volunteers was approved by the Cardiff University School of Medicine REC (18/4). All participants provided written informed consent for the collection of peripheral blood samples and subsequent analyses in accordance with the principles of the Declaration of Helsinki.

### Cell culture and in vitro stimulation assays

Human PBMCs were isolated via density gradient centrifugation using Histopaque-1077 (Merck Life Sciences, Gillingham, UK) and cryopreserved in FBS containing 10% DMSO (Merck Life Sciences). Human PBMCs were thawed and rested overnight for ∼24 h in R10 media (RPMI 1640 medium supplemented with 10% FBS, 100 U/ml penicillin, 100 μg/ml streptomycin, and 2 mM L-glutamine; Thermo Fisher Scientific, Altrincham, UK) at 37 °C and in 5% CO_2_ prior to being seeded in 24-well or 96-well culture plates for in vitro stimulation assays. Cell Activation Cocktail without Brefeldin A (BioLegend, London, UK) was used to deliver 25 ng/ml phorbol-12-myristate 13-acetate (PMA) and 500 ng/ml ionomycin to cultured PBMCs for 6 h at 37 °C. Recombinant forms of SEA, SEB, or TSST-1 from *S. aureus* (Toxin Technology, Sarasota, FL, USA), each at a dose of 100 ng/ml to align with previous work,^19^^,^^70–72^ were used to stimulate PBMCs for 24 h, unless otherwise stated. Unstimulated cells were used as negative controls. In mechanistic experiments, human PBMCs were preincubated for 30 min with 2 μg/ml anti-human IL-12 (clone 24910; BioTechne, Minneapolis, MN, USA) or 2 μg/ml mouse IgG_1_ (clone MOPC-21; BioLegend). In functional experiments, anti-CD107a–BV421 (clone H4A3; BioLegend) was added to the cultures with PMA/ionomycin or each individual SAg,^73^ and protein transport was blocked for the final 6 h with GolgiPlug (1:1000; BD Biosciences, Franklin Lakes, NJ, USA) and GolgiStop (1:1500; BD Biosciences).

### Flow cytometry

Cells were washed in Dulbecco’s PBS (Thermo Fisher Scientific), labeled for 15 to 30 min at room temperature (RT) with Zombie Aqua Fixable Viability Kit (BioLegend) for live/dead cell exclusion. Surface stains were performed for 30 min at 4 °C using combinations of the following directly conjugated mAbs: (i) anti-CD3–APC/Fire 750 (clone SK7), anti-CD3–PerCP (clone SK7), anti-CD16–BV711 (clone 3G8), anti-CD56–BV605 (clone HCD56), anti-CD56–BV711 (clone HCD56), anti-CD57–PE/Dazzle 594 (clone QA17A04), anti-CD69–APC (clone FN50), anti-CD69–BV421 (clone FN50), anti-NKG2C–PE (clone S19005E), anti-NKp30–APC/Fire 750 (clone P30-15), anti-Siglec-7–APC/Fire 750 (clone 6-434), and anti-TIGIT-BV421 (clone A15153G) from BioLegend; (ii) anti-CD14 V500 (clone M5E2) and anti-CD19 V500 (clone HIB19) from BD Biosciences; and (iii) anti-NKG2A-VioBright FITC (clone REA110) from Miltenyi Biotec. Cytosolic/intranuclear expression of the proliferation marker Ki67 was detected using anti-Ki67–BV421 (clone B56; BD Biosciences) in conjunction with an eBioscience FOXP3/FOXP3 Transcription Factor Staining Buffer Kit (Thermo Fisher Scientific). Intracellular cytokines were exposed using a Cytofix/Cytoperm Plus Fixation/Permeabilization Solution Kit (BD Biosciences) or eBioscience FOXP3/Transcription Factor Staining Buffer Kit (Thermo Fisher Scientific) and stained for 30 min at 4 °C with combinations of the following directly conjugated mAbs: anti-IFN-γ–APC (clone B27), anti-IFN-γ–PE/Cyanine7 (clone B27), and anti-TNF-α–APC (clone MAb11) from BioLegend. All flow cytometry panels were validated using individually stained BD CompBeads anti-mouse Ig, κ/Negative Control Compensation Particles (BD Biosciences), or MACS anti-REA Comp Bead kit (Miltenyi Biotec). Data were acquired using an Attune NxT flow cytometer (Thermo Fisher Scientific) and analyzed using FlowJo software version 10.10 (FlowJo LLC, Ashland, OR, USA).

### IL-12p70–specific ELISA

Plasma was collected from patients with *S. aureus* or *E. coli* bacteremia and healthy controls during the density gradient centrifugation of peripheral blood using Histopaque-1077. Aliquots were prepared and stored at -80°C until further use. IL-12p70 levels were quantified using a commercial ELISA kit (Mabtech, Nacka Strand, Sweden). Nunc MaxiSorp 96-well plates (BioLegend) were coated with an anti-IL-12p40 capture antibody (2 μg/ml; Mabtech; clone MT86/221) and incubated overnight at 4 °C. Following incubation, plates were blocked with PBS supplemented with 0.1% BSA and 0.05% Tween-20 (Merck Life Sciences) for 1 h at RT to reduce nonspecific binding. Following this, coated plates were washed 3 times with PBS containing 0.05% Tween-20 before undiluted plasma samples and recombinant IL-12p70 protein (serially diluted from 1 ng/ml) were added in duplicate and incubated for 2 h at RT. After further washing, plates were incubated for 1 h at RT with a biotin-conjugated anti-IL-12p70 detection antibody (1 μg/ml; Mabtech clone MT704) before the process was repeated using streptavidin conjugated to alkaline phosphatase (Mabtech; 1:1000 dilution of stock protein provided). Following further washing with PBS containing 0.05% Tween-20, Para-nitrophenylphosphate (Mabtech) was added to each well and incubated for 60 min at RT to develop in the absence of light. Optical densities were read at 405 nm using the CLARIOstar microplate reader (BMG Labtech). Samples were blinded to eliminate operator bias. Standard curves were plotted using a nonlinear regression model on Prism v10.1.0 (GraphPad Software, La Jolla, CA, USA), with concentrations calculated in reference to the curve. All recombinant IL-12p70 protein standard and human plasma sample aliquots were thawed immediately prior to use.

### Statistical analysis

Differences between 2 groups were evaluated using an unpaired *t*-test while differences between 3 or more groups were evaluated using a 1-way ANOVA with Tukey post hoc test in Prism v10.1.0 (GraphPad). Significance was assigned at *P* < 0.05.

## Results

### Circulating CD57^−^ NKG2A^+^ NK cells increase in frequency *in vivo* during *S. aureus* bacteremia

**Figure 1.**
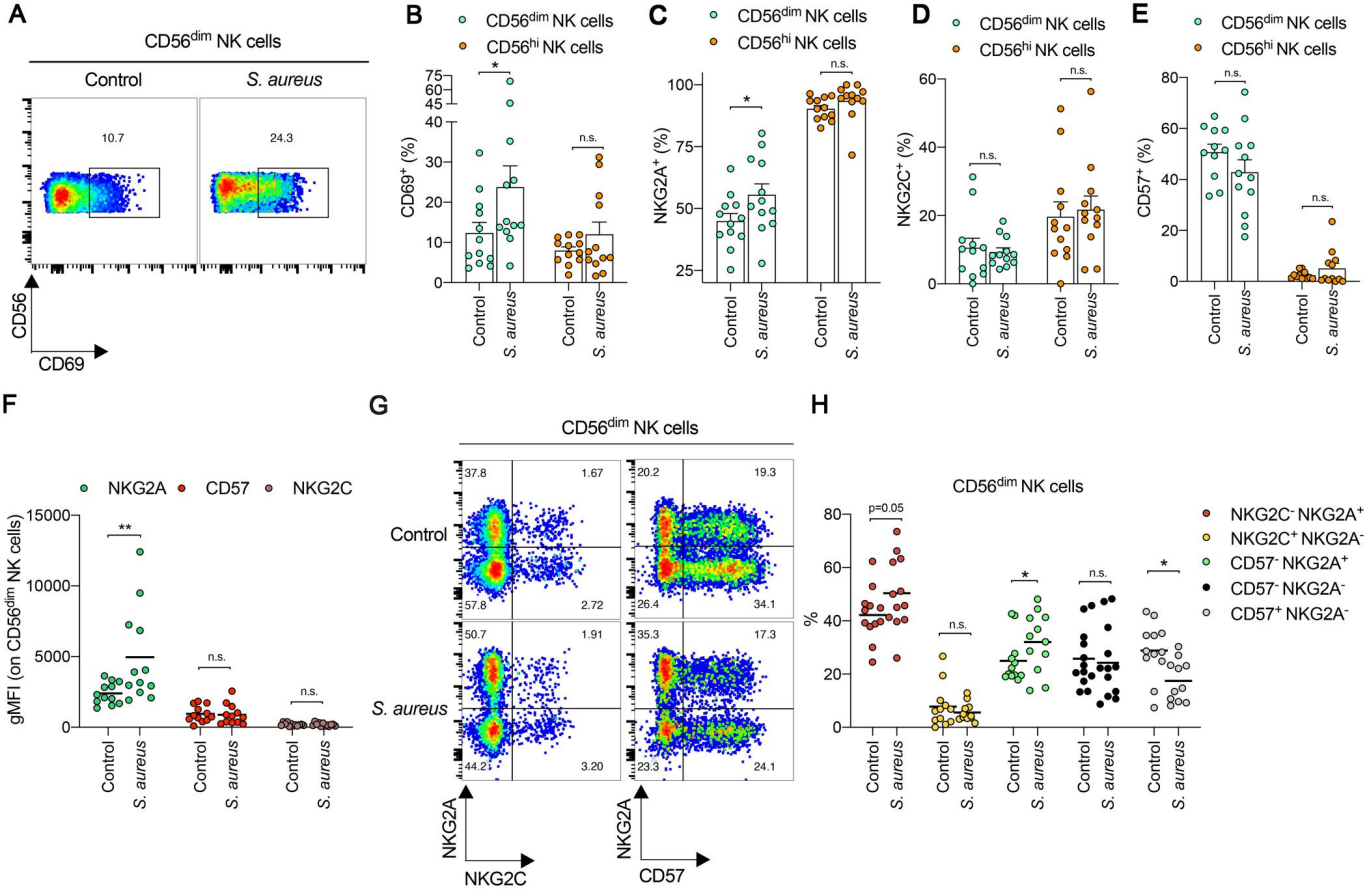
Circulating CD57^−^ NKG2A^+^ NK cells expand *in vivo* during *S. aureus* bacteremia in humans. (A) Representative flow cytometry plots depicting expression levels of CD69 on CD56^dim^ NK cells from human PBMCs collected from healthy controls and patients hospitalized with *S. aureus* bacteremia. Frequency of CD69^+^ (B), NKG2A^+^ (C), NKG2C^+^ (D), or CD57^+^ (E) cells among CD56^dim^ (green filled circles) or CD56^hi^ (orange filled circles) NK cells from human PBMCs collected from healthy controls and patients hospitalized with *S. aureus* bacteremia. Each dot represents 1 control or patient. Data are shown as mean ± SEM. *P < 0.05; unpaired *t*-test. n.s. = not significant. (F) Scatter dot plots depicting geometric mean fluorescence intensity (gMFI) of NKG2A (green filled circles), CD57 (red filled circles), or NKG2C (dark pink filled circles) expression on CD56^dim^ NK cells from human PBMCs collected from healthy controls and patients hospitalized with *S. aureus* bacteremia. Each dot represents 1 donor. Line indicates mean value. **P < 0.01; unpaired *t*-test. n.s. = not significant. (G) Representative flow cytometry plots depicting expression levels of NKG2A vs NKG2C or NKG2A vs CD57 on CD56^dim^ NK cells from human PBMCs collected from healthy controls and patients hospitalized with *S. aureus* bacteremia. (H) Scatter dot plot showing the frequency of NKG2C^−^ NKG2A^+^ (red filled circles), NKG2C^+^ NKG2A^−^ (yellow filled circles), CD57^−^ NKG2A^+^ (green filled circles), CD57^−^ NKG2A^−^ (black filled circles), or CD57^+^ NKG2A^−^ (gray filled circles) subsets among CD56^dim^ NK cells from human PBMCs collected from healthy controls and patients hospitalized with *S. aureus* bacteremia. Each dot represents 1 control or patient. Line indicates mean value. *P < 0.05; unpaired *t*-test. n.s. = not significant.

To study the NK cell response to *S. aureus*, we used multiparameter flow cytometry panels to identify and phenotype (Fig. S1) NK cell subsets (e.g. CD56^dim^ and CD56^hi^ NK cells) in PBMCs collected from adult patients hospitalized with *S. aureus* bacteremia (*n *= 12). PBMCs from sex-matched healthy controls (*n *= 12) were collected for comparison (Table 1). When assessing global NK cell frequency within all NK cells and all CD3^−^ CD14^−^ CD19^−^ lymphocytes, patients with *S. aureus* bacteremia had lower frequencies of CD56^dim^ NK cells compared to healthy controls (Figure S2A, B). Using CD69 as a marker of NK cell activation, we saw a significant increase in percentage of CD69^+^ cells among all CD56^dim^ NK cells, but not CD56^hi^ NK cells (Fig. 1A, B). Using NKG2A, NKG2C, and CD57 as markers of NK cell differentiation states, we found there was a significant increase in the percentage of NKG2A^+^ CD56^dim^ NK cells in patients with *S. aureus* bacteremia (Fig. 1C), but not CD56^hi^ NK cells. In contrast, there was no significant change in the percentage of NKG2C^+^ or CD57^+^ CD56^dim^ or CD56^hi^ NK cells in patients with *S. aureus* bacteremia (Fig. 1D, E), although there was a trend for lower proportions of CD57^+^ CD56^dim^ NK cells in these patients. In support of this phenotypic change in NK cells, NKG2A expression levels were significantly higher on CD56^dim^ NK cells from patients with *S. aureus* bacteremia, compared to healthy controls, while no change in CD57 or NKG2C expression was observed (Fig. 1F). When segregating CD56^dim^ NK cells based on NKG2A, NKG2C, and/or CD57 expression, we found that patients with *S. aureus* bacteremia displayed a significant increase in the percentage of CD57^−^ NKG2A^+^ NK cells compared to healthy controls, with a concurrent significant reduction in CD57^+^ NKG2A^−^ NK cells (Fig. 1G, H). These changes in CD57^−^ NKG2A^+^ and CD57^+^ NKG2A^−^ NK cells in *S. aureus* patients were NK cell specific as they were not recapitulated in CD56^+^ T cells (Figure S3), which includes invariant-like subsets such as NKT cells and mucosal-associated invariant T (MAIT) cells.^74^ In these CD56^+^ T cells, the percentage of CD57^−^ NKG2A^+^ cells was no different to that seen in healthy controls while CD57^+^ NKG2A^−^ cells trended higher in number, albeit without reaching significance (Fig. S3). IL-12 is known to upregulate NKG2A expression on NK cells.^75^^,^^76^ However, plasma IL-12p70 levels were not significantly different in patients with *S. aureus* bacteremia compared to healthy controls (Figure S4A), despite a positive correlation between plasma IL-12p70 levels and the percentage of NKG2C^−^ NKG2A^+^ NK cells in patients with *S. aureus* (Fig. S4B).

**Table 1.** Baseline demographics of adult patients with *S. aureus* or *E. coli* bacteremia compared to healthy controls.

Characteristic	*S. aureus* bacteremia (*n *= 12)	*E. coli* bacteremia (*n *= 12)	Healthy controls (*n *= 12)
Age, y			
Median	63.6	66.8	65.4
Range	27.6–93	31.6–74.9	33.5–73.8
Sex			
Female	7	8	7
Male	5	4	5
SOFA score			
<2	4	4	–
≥2	8	8	–
CRP at admission, mg/L			
Median	228	168.5	–
Range	12–383	51–433	–
Time of sampling (days postdiagnosis)			
Median	3.0	3.0	–
Range	2–5	2–5	–

Diagnosis indicates positive blood culture for *S. aureus* or *E. coli.*

Abbreviations: CRP, C-reactive protein; SOFA, Sequential Organ Failure Assessment.

To validate whether these NK cell–specific changes, most notably in increased frequency of CD57^−^ NKG2A^+^ subsets, were a specific outcome of *S. aureus* bacteremia rather than a generalized immune response to a bacterial pathogen in the bloodstream, we next examined phenotypic alterations in NK cells in patients hospitalized with *E. coli* bacteremia. In contrast to patients with *S. aureus* bacteremia, there was no increase in the percentage of CD69^+^ CD56^dim^ NK cells in patients with *E. coli* bacteremia (Fig. S5A) compared to healthy controls. Furthermore, the percentage of CD57^−^ NKG2A^+^ or CD57^+^ NKG2A^−^ NK cells did not change in patients with *E. coli* bacteremia (Fig. S5B), nor were any alterations in NKG2A expression on CD56^dim^ NK cells (Fig. S5C), or plasma IL-12p70 levels, observed (Fig. S5D). These data suggest that the presence of *S. aureus* in the bloodstream, in contrast to *E. coli*, was driving an increase in NK cell activation while also promoting an increase in the frequency of CD57^−^ NKG2A^+^ NK cell subsets.

**Figure 2.**
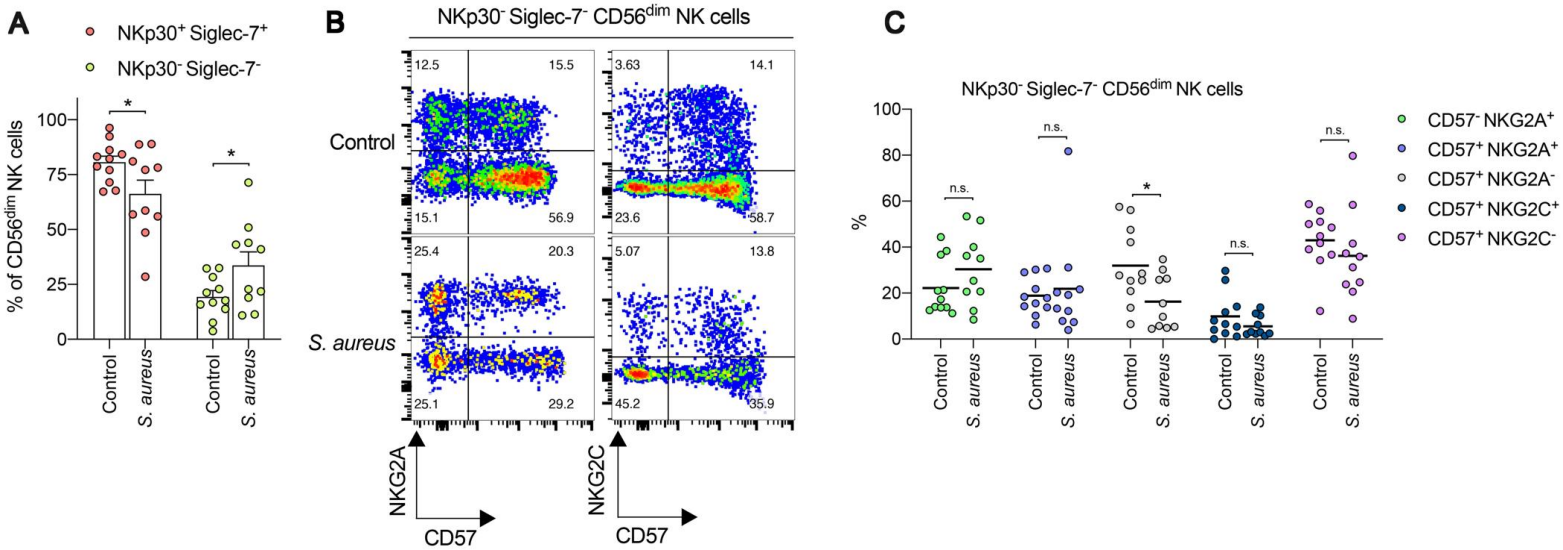
Patients with *S. aureus* bacteremia display increased frequencies of NKp30^−^ Siglec-7^−^ NK cells. (A) Frequency of NKp30^+^ Siglec-7^+^ (light red filled circles) or NKp30^−^ Siglec-7^−^ (light green filled circles) among CD56^dim^ NK cells from human PBMCs collected from healthy controls and patients hospitalized with *S. aureus* bacteremia. Each dot represents 1 control or patient. Data are shown as mean ± SEM. *P < 0.05; unpaired *t*-test. (B) Representative flow cytometry plots depicting expression levels of NKG2A or NKG2C vs CD57 on NKp30^−^ Siglec-7^−^ CD56^dim^ NK cells from human PBMCs collected from healthy controls and patients hospitalized with *S. aureus* bacteremia. (C) Scatter dot plot showing the frequency of CD57^−^ NKG2A^+^ (green filled circles), CD57^+^ NKG2A^+^ (light blue filled circles), CD57^+^ NKG2A^−^ (gray filled circles), CD57^+^ NKG2C^+^ (dark blue filled circles), or CD57^+^ NKG2C^−^ (purple filled circles) subsets among NKp30^−^ Siglec-7^−^ CD56^dim^ NK cells from human PBMCs collected from healthy controls and patients hospitalized with *S. aureus* bacteremia. Each dot represents 1 control or patient. Line indicates mean value. *P < 0.05; unpaired *t*-test. n.s. = not significant.

Adaptive NK cells typically represent highly differentiated CD57^+^ NKG2C^+^ populations that preferentially lack expression of NKp30 and also sialic acid–binding Ig-like lectin 7 (Siglec-7), which mediates an inhibitory effect on NK cell cytotoxicity.^46^^,^^47^^,^^77–79^ Our data demonstrated a significant reduction in CD57^+^ NKG2A^−^ CD56^dim^ NK cells in patients with *S. aureus* bacteremia (Fig. 1G, H), which include NK cells with a CD57^+^ NKG2C^+^ phenotype. Accordingly, this might suggest that adaptive NK cell phenotypes (e.g. NKp30^−^ Siglec-7^−^) could also be reduced in patients with *S. aureus* bacteremia. However, we found there was a significant increase in the frequency of NKp30^−^ Siglec-7^−^ CD56^dim^ NK cells in patients with *S. aureus* bacteremia, compared to healthy controls (Fig. 2A). This effect was not observed in CD56^dim^ NK cells from patients with *E. coli* bacteremia (Figure S5E). After pre-gating on NKp30^−^ Siglec-7^−^ CD56^dim^ NK cells, we did not observe a significant change in the percentage of CD57^+^ NKG2C^+^ subsets in patients with *S. aureus* bacteremia, compared to healthy controls (Fig. 2B, C). No difference in the proportions of CD57^+^ NKG2A^+^ or CD57^+^ NKG2C^−^ cell subsets were seen between both groups (Fig. 2B, C). Instead, we saw a significant reduction in the percentage of CD57^+^ NKG2A^−^ subsets in patients with *S. aureus* bacteremia along with a concurrent rise in the proportion of CD57^−^ NKG2A^+^ NK cell subsets (Fig. 2B, C), in agreement with our data on all CD56^dim^ NK cells.

Collectively, these data show that circulating CD57^−^ NKG2A^+^ NK cells increase in frequency in patients with *S. aureus* bacteremia, while subsets lacking NKp30 and Siglec-7 increase in percentage upon systemic infection without affecting adaptive CD57^+^ NKG2C^+^ NK cell phenotypes.

### SAgs promote the expansion of CD57^−^ NKG2A^+^ NK cells

**Figure 3.**
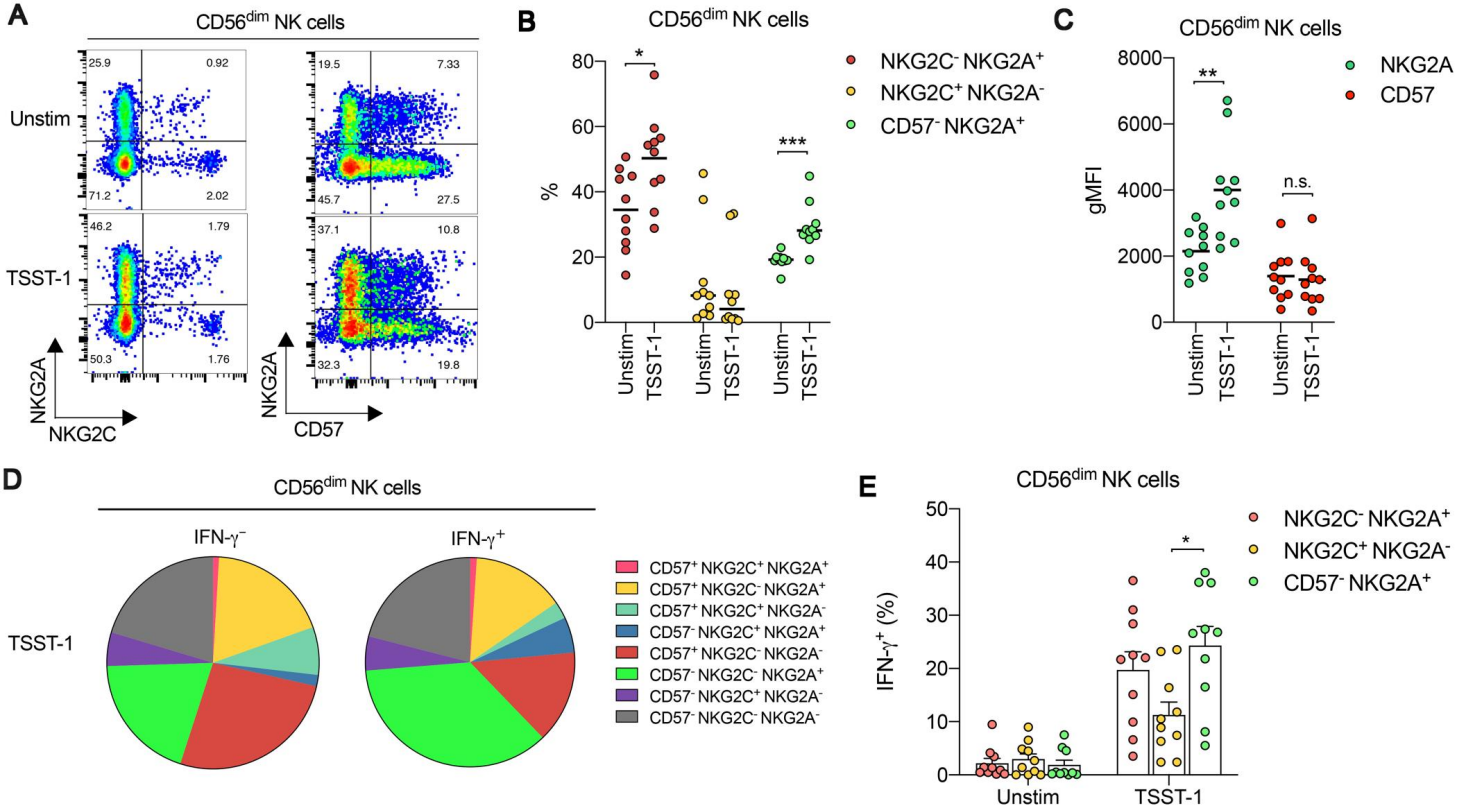
TSST-1 promotes the expansion of CD57^−^ NKG2A^+^ NK cells. (A) Representative flow cytometry plots depicting expression levels of NKG2A vs NKG2C or NKG2A vs CD57 on CD56^dim^ NK cells from human PBMCs collected from healthy controls that were cultured in medium alone (Unstim) or stimulated with TSST-1 for 24 h. (B) Scatter dot plot showing the frequency of NKG2C^−^ NKG2A^+^ (red filled circles), NKG2C^+^ NKG2A^−^ (yellow filled circles), or CD57^−^ NKG2A^+^ (green filled circles) subsets among CD56^dim^ NK cells from human PBMCs collected from healthy controls that were cultured in medium alone (Unstim) or stimulated with TSST-1 for 24 h. Each dot represents 1 donor. Line indicates mean value. *P < 0.05, ***P < 0.001; unpaired *t*-test. (C) Scatter dot plots depicting geometric mean fluorescence intensity (gMFI) of NKG2A (green filled circles) or CD57 (red filled circles) expression on CD56^dim^ NK cells from human PBMCs collected from healthy controls that were cultured in medium alone (Unstim) or stimulated with TSST-1 for 24 h. Each dot represents 1 donor. Line indicates mean value. **P < 0.01; Mann-Whitney *U*-test. n.s. = not significant. (D) Pie charts depicting the proportion of IFN-γ^−^ or IFN-γ^+^ CD56^dim^ NK cells that are made up of subsets that express combinations of CD57, NKG2A, and/or NKG2C from human PBMCs that were stimulated with TSST-1 for 24 h. Data are concatenated from *n =* 6 individual donors. (E) Frequency of IFN-γ^+^ cells among NKG2C^−^ NKG2A^+^ (light red filled circles), NKG2C^+^ NKG2A^−^ (yellow filled circles), or CD57^−^ NKG2A^+^ (green filled circles) CD56^dim^ NK cells from human PBMCs collected from healthy controls that were cultured in medium alone (Unstim) or stimulated with TSST-1 for 24 h. Each dot represents 1 donor. Data are shown as mean ± SEM; *P < 0.05; 1-way ANOVA with Tukey post hoc test.

Virulence factors encoded by *S. aureus* are capable of targeting NK cells and/or modifying their functionality.^65–69^ SAgs, which typically target T cells and human leukocyte antigen (HLA) class II^+^ APCs, can indirectly activate human NK cells, which produce more IFN-γ in response to SAg stimulation than innate-like T cell subsets such as MAIT cells.^65^^,^^66^ SAgs also promote the production of IL-12 from myeloid cells, which is important for inducing IFN-γ production, rather than cytotoxic granule release, from human NK cells.^66^^,^^80^^,^^81^ Given that IL-12 also upregulates NKG2A expression on both NK cells^75^^,^^76^ and T cells,^82^ we next examined whether candidate SAgs from *S. aureus* could promote the formation of CD57^−^ NKG2A^+^ NK cells *in vitro*. Using PBMCs from healthy donors, stimulation with recombinant forms of SEA, SEB, and TSST-1 (24 h) produced significant increases in the percentage of IFN-γ^+^, CD107a^+^, and CD69^+^ cells among CD56^dim^ NK cells (Figure S6A–D). Indeed, the degree of activation was highest in CD56^hi^ NK cells, while that observed in CD56^dim^ NK cells was comparable to CD56^+^ T cells and higher than that seen in more conventional, CD56^−^ T cells, in agreement with published data.^66^ Importantly though, all three SAgs promoted an increase in the percentage of NKG2C^−^ NKG2A^+^ CD56^dim^ NK cells, but also CD57^−^ NKG2A^+^ populations (Figs. 3A, B, and S7A, B). These effects were most evident following TSST-1 stimulation, which yielded significant increases in the percentage of NKG2C^−^ NKG2A^+^ and CD57^−^ NKG2A^+^ CD56^dim^ NK cells (Fig. 3B) but also in the expression levels of NKG2A on the surface of CD56^dim^ NK cells (Fig. 3C). This effect was confined to CD56^dim^ NK cells as all three SAgs yielded very little change in the percentage of NKG2C^−^ NKG2A^+^ CD56^hi^ NK cells (Figure S7C) while the proportion of CD57^−^ NKG2A^+^ cells within CD56^hi^ NK cells even reduced upon stimulation (Fig. S7D). The largest proportion of IFN-γ^+^ CD56^dim^ NK cells in response to SAg stimulation was confined to the CD57^−^ NKG2C^−^ NKG2A^+^ subset (Figs. 3D and S8). Furthermore, the percentage of TSST-1–induced IFN-γ^+^ cells was significantly higher in CD57^−^ NKG2A^+^ CD56^dim^ NK cells, compared to NKG2C^+^ NKG2A^−^ or CD57^+^ NKG2A^+^ NK cell subsets (Fig. 3E).

**Figure 4.**
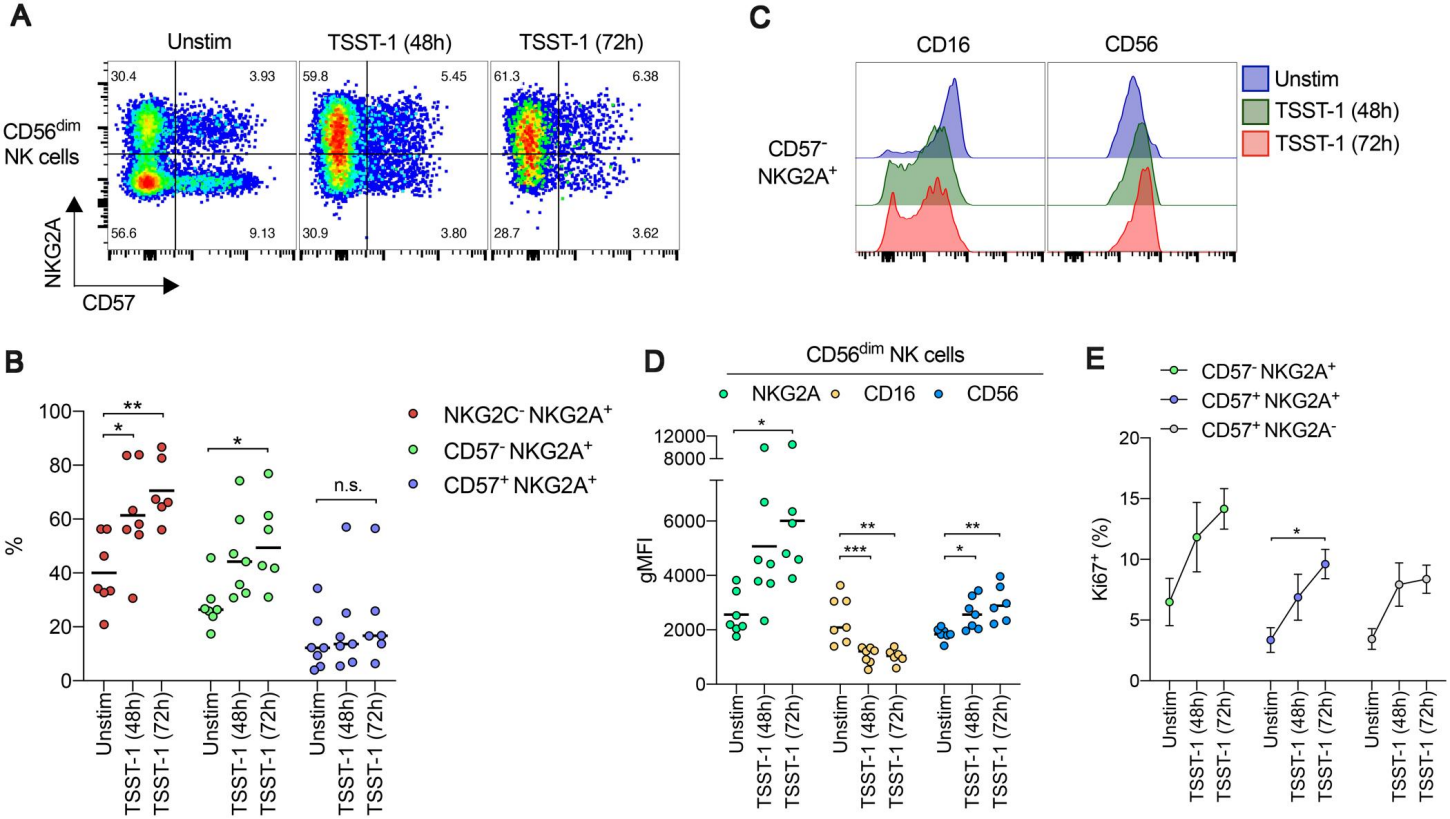
TSST-1–mediated alterations in NKG2A^+^ NK cells are more pronounced over time. (A) Representative flow cytometry plots depicting expression levels of NKG2A vs CD57 on CD56^dim^ NK cells from human PBMCs collected from healthy controls that were cultured in medium alone (Unstim) or stimulated with TSST-1 for 48 h or 72 h. (B) Scatter dot plots showing the frequency of NKG2C^−^ NKG2A^+^ (red filled circles), CD57^−^ NKG2A^+^ (green filled circles), or CD57^+^ NKG2A^+^ (light blue filled circles) subsets among CD56^dim^ NK cells from human PBMCs collected from healthy controls that were cultured in medium alone (Unstim) or stimulated with TSST-1 for 48 h or 72 h. Each dot represents 1 donor. Line indicates mean value. *P < 0.05, **P < 0.01; 1-way ANOVA with Tukey post hoc test. (C) Representative overlay histograms showing expression of CD16 or CD56 on CD57^−^ NKG2A^+^ CD56^dim^ NK cells from human PBMCs collected from healthy controls that were cultured in medium alone (Unstim) or stimulated with TSST-1 for 48 h or 72 h. (D) Scatter dot plots depicting geometric mean fluorescence intensity (gMFI) of NKG2A (green filled circles), CD16 (yellow filled circles), or CD56 (blue filled circles) expression on CD56^dim^ NK cells from human PBMCs collected from healthy controls that were cultured in medium alone (Unstim) or stimulated with TSST-1 for 48 h or 72 h. Each dot represents 1 donor. Line indicates mean value. *P <0.05, **P < 0.01, ***P < 0.001; 1-way ANOVA with Tukey post hoc test. (E) Frequency of Ki67^+^ cells among CD57^−^ NKG2A^+^ (green filled circles), CD57^+^ NKG2A^+^ (blue filled circles), or CD57^+^ NKG2A^−^ (gray filled circles) CD56^dim^ NK cells from human PBMCs collected from healthy controls that were cultured in medium (*n =* 7) alone (Unstim) or stimulated with TSST-1 for 48 h (*n =* 7) or 72 h (*n =* 6). Data are shown as mean ± SEM; *P < 0.05; 1-way ANOVA with Tukey post hoc test.

To further explore the effects of SAg stimulation on CD57^−^ NKG2A^+^ CD56^dim^ NK cell subsets, we examined longer stimulation time periods using TSST-1 as a candidate SAg to determine whether such phenotypic polarization was more pronounced over time. Indeed, the percentage of CD57^−^ NKG2A^+^ or NKG2C^−^ NKG2A^+^ CD56^dim^ NK cells continued to increase significantly following a period of 48 h or 72 h stimulation (Fig. 4A, B). In line with these observations, NKG2A expression levels on the surface of CD56^dim^ NK cells significantly increased further following 48 h or 72 h stimulation. Furthermore, CD16 expression levels were significantly downregulated by TSST-1 stimulation on CD56^dim^ NK cells and CD57^−^ NKG2A^+^ subsets, while CD56 expression also increased significantly over time with stimulation (Fig. 4C, D). These changes were consistent with the notion of TSST-1 expanding a less differentiated NK cell phenotype upon stimulation. Additionally, the highest expression frequencies of the proliferation marker Ki67 were seen in CD57^−^ NKG2A^+^ CD56^dim^ NK cells after 72 h stimulation with TSST-1 (Fig. 4E).

Collectively, these data show that TSST-1 stimulation promotes the expansion of NKG2A^+^ NK cells within human PBMCs, favoring highly proliferative, IFN-γ–producing CD57^−^ NKG2A^+^ subsets while simultaneously driving a phenotypic shift toward this population.

### TSST-1–mediated promotion of a CD57^−^ NKG2A^+^ NK cell phenotype appears to be IL-12 independent

**Figure 5.**
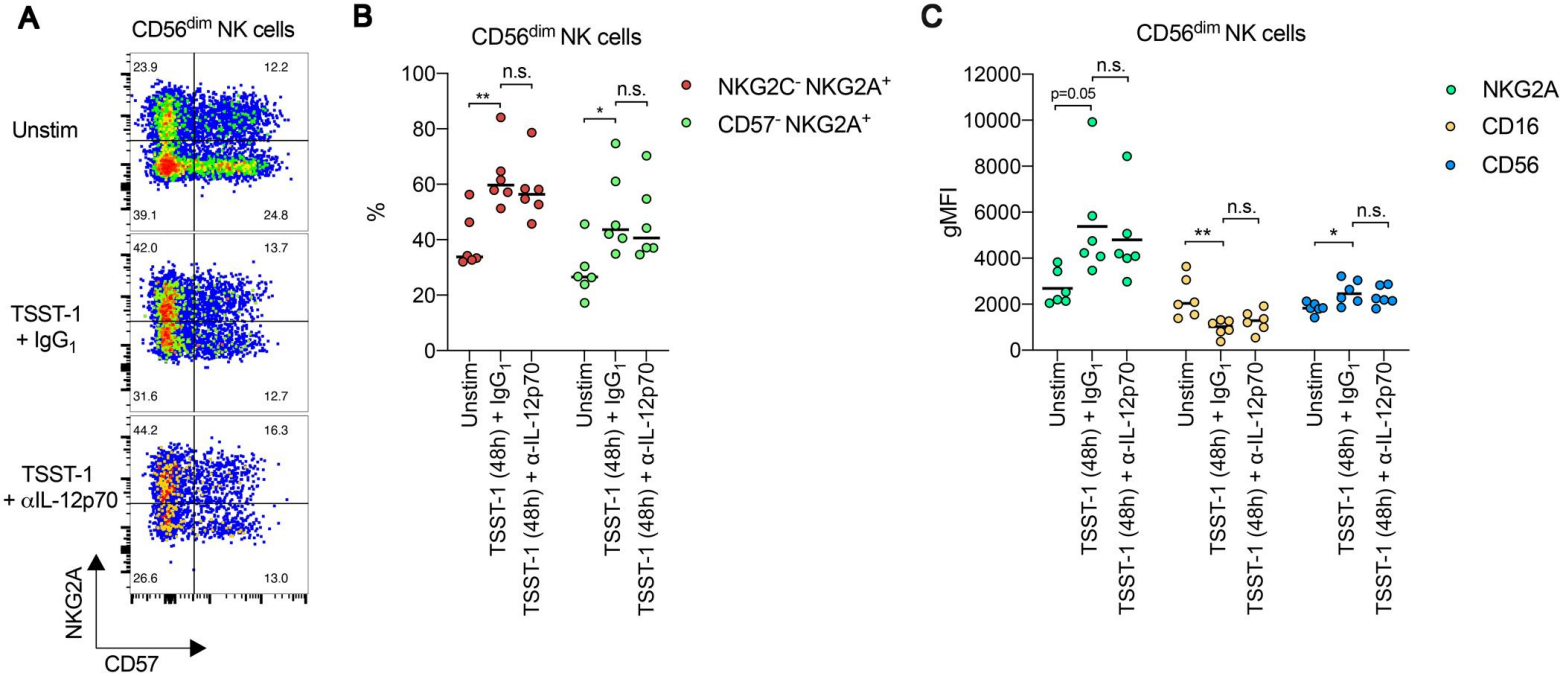
TSST-1–mediated polarization toward a CD57^−^ NKG2A^+^ NK cell phenotype involves an IL-12–independent mechanism. (A) Representative flow cytometry plots depicting expression levels of NKG2A vs CD57 on CD56^dim^ NK cells from human PBMCs collected from healthy controls that were cultured in medium alone (Unstim) or pretreated with anti-human IL-12p70 or an isotype control antibody (IgG_1_) before being stimulated with TSST-1 for 48 h. (B) Scatter dot plot showing the frequency of NKG2C^−^ NKG2A^+^ (red filled circles) or CD57^−^ NKG2A^+^ (green filled circles) subsets among CD56^dim^ NK cells from human PBMCs collected from healthy controls that were cultured in medium alone (Unstim) or pretreated with anti-human IL-12p70 or an isotype control antibody (IgG_1_) before being stimulated with TSST-1 for 48 h. Each dot represents 1 donor. Line indicates mean value. (C) Scatter dot plots depicting geometric mean fluorescence intensity (gMFI) of NKG2A (green filled circles), CD16 (yellow filled circles), or CD56 (blue filled circles) expression on CD56^dim^ NK cells from human PBMCs collected from healthy controls that were cultured in medium alone (Unstim) or pretreated with anti-human IL-12p70 or an isotype control antibody (IgG_1_) before being stimulated with TSST-1 for 48 h. Each dot represents 1 donor. Line indicates mean value. *P < 0.05, **P < 0.01; 1-way ANOVA with Tukey post hoc test.

IL-12 is known to upregulate NKG2A expression on NK cells.^75^^,^^76^ Thus, we next used an anti-IL-12p70 neutralizing antibody to determine if the increased percentage of CD57^−^ NKG2A^+^ CD56^dim^ NK cells elicited after TSST-1 stimulation of PBMCs involved an IL-12–dependent mechanism. IL-12 blockade produced a trend toward a lower percentage of IFN-γ^+^ CD56^dim^ NK cells elicited after 48 h stimulation with TSST-1 (Fig. S9A, B), in line with published data.^66^ However, neutralization of IL-12 appeared to have no discernible effect on the expansion of CD57^−^ NKG2A^+^ or NKG2C^−^ NKG2A^+^ CD56^dim^ NK cells that occurred after 48 h of TSST-1 stimulation (Fig. 5A, B). In line with these observations, we saw no effect on NKG2A expression changes on the surface of CD56^dim^ NK cells by TSST-1 (Fig. 5C). Furthermore, TSST-1–mediated changes in CD16 and CD56 expression were also IL-12 independent (Fig. 5C). These data suggest that while IL-12 is an important factor in SAg-induced IFN-γ production from CD56^dim^ NK cells, it appears to not be involved in driving CD56^dim^ NK cells toward a CD57^−^ NKG2A^+^ NK cell phenotype.

### CD56^dim^ NK cells in human patients with *S. aureus* bacteremia are more polyfunctional

**Figure 6.**
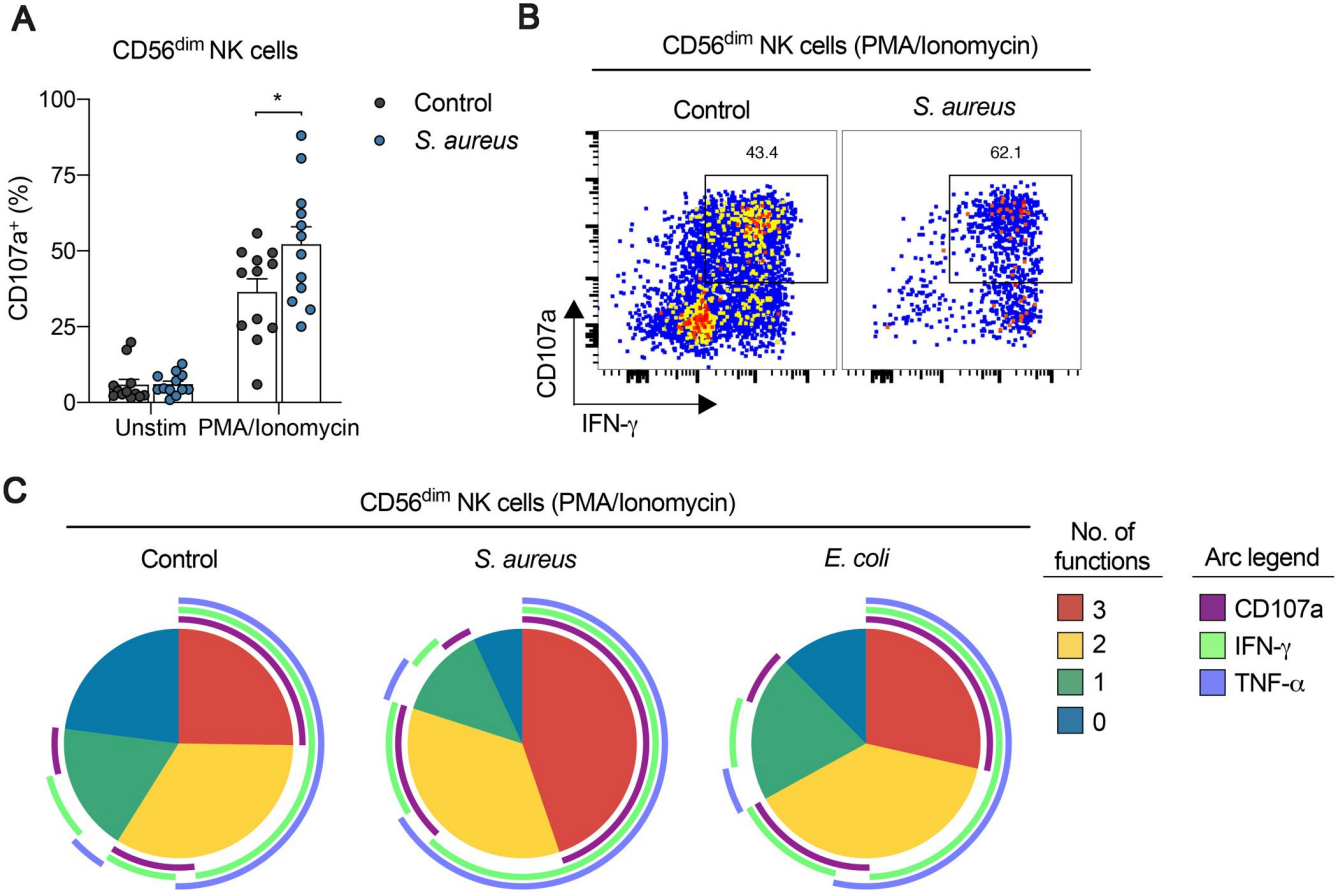
CD56^dim^ NK cells in human patients with *S. aureus* bacteremia are more polyfunctional, yielding more effector cytokines upon stimulation. (A) Frequency of CD107a^+^ cells among CD56^dim^ NK cells from human PBMCs collected from healthy controls (black filled circles) and patients hospitalized with *S. aureus* bacteremia (blue filled circles) cultured in medium alone (Unstim) or with PMA/ionomycin for 6 h. Data are shown as mean ± SEM; *P < 0.05; unpaired *t*-test. (B) Representative flow cytometry plots depicting expression levels of CD107a and IFN-γ among CD56^dim^ NK cells from human PBMCs collected from healthy controls and patients hospitalized with *S. aureus* bacteremia cultured with PMA/ionomycin for 6 h. (C) Functional profiles of CD56^dim^ NK cells from human PBMCs collected from healthy controls and patients hospitalized with *S. aureus* or *E. coli* bacteremia stimulated with PMA/ionomycin for 6 h. Pie chart segments from concatenated data represent the fraction of cells displaying the indicated number of functions (key). Arcs denote individual functions (key).

Peripheral blood NKG2A^+^ NK cells are effective cytokine producers, and differentiation toward CD57^+^ NKG2C^+^ populations generates NK cells that are less cytokine responsive but with more specialized functions such as receptor-driven cytotoxicity.^32^^,^^36–38^ Our data demonstrate that CD57^−^ NKG2A^+^ NK cells increase in frequency during *S. aureus* bacteremia. As such, we hypothesized that *S. aureus* bacteremia may generate NK cells with more degranulating and cytokine-producing capacity. To test this, we stimulated PBMCs collected from healthy controls and patients with *S. aureus* bacteremia with PMA/ionomycin and measured the percentage of CD56^dim^ NK cells that degranulated (i.e. based on surface mobilization of CD107a^83^) and produced IFN-γ and/or TNF-α. We found that PMA/ionomycin stimulation yielded a significant increase in CD107a^+^ CD56^dim^ NK cells from patients with *S. aureus* bacteremia, compared to healthy controls (Fig. 6A). A higher proportion of CD107a^+^ NK cells in patients with *S. aureus* were IFN-γ^+^ (Fig. 6B), suggesting that patients with *S. aureus* bacteremia may possess more polyfunctional NK cells, compared to healthy controls. Indeed, using Boolean gating and Simplified Presentation of Incredibly Complex Evaluations software^84^ to assess the degree of NK cell polyfunctionality, patients with *S. aureus* bacteremia displayed a higher proportion of polyfunctional CD56^dim^ NK cells and fewer unresponsive CD56^dim^ NK cells (CD107a^−^ IFN-γ^−^ TNF-α^−^) compared to healthy controls (Fig. 6C).

Collectively, these results demonstrate that CD56^dim^ NK cells in patients with *S. aureus* bacteremia are functionally more responsive to stimulation, yielding more polyfunctional NK cells, compared to healthy controls.

## Discussion

In this study, we observed an increase in the frequency of CD57^−^ NKG2A^+^ NK cells in patients with *S. aureus* bacteremia compared to healthy controls and found that NK cells from these patients were functionally more responsive to stimulation, displaying evidence of being more polyfunctional and, thus, being capable of producing more effector cytokines, such as IFN-γ. Accordingly, our data suggest that invasive *S. aureus* infection *in vivo* promotes a phenotypic shift toward NK cell subsets with greater IFN-γ–producing capacity and notionally agrees with the concept that NK cells may promote inflammation-mediated disease pathogenesis during *S. aureus* bacteremia. As far as we are aware, our study is the first to demonstrate this phenotypic alteration toward CD57^−^ NKG2A^+^ NK cells in this patient group and is consistent with studies in mouse models of *S. aureus* infection, suggesting that NK cell–released IFN-γ may promote immune pathogenesis. In contrast, we did not observe the same alteration of CD57^−^ NKG2A^+^ NK cells in patients with *E. coli* bacteremia, despite mouse models indicating that NK cells may also be immunopathogenic during systemic *E. coli* infection.^57^ This suggests that *S. aureus* itself may be directly responsible for triggering this change in the frequency of CD57^−^ NKG2A^+^ NK cells in patients. Indeed, our data show that SAgs from *S. aureus*, namely TSST-1, can upregulate NKG2A expression on NK cells and promote the expansion of highly proliferative, IFN-γ–producing CD57^−^ NKG2A^+^ subsets.

SAgs may not be the sole driver behind these phenotypic changes as *S. aureus* also encodes pore-forming toxins that affect NK cell behavior. In this regard, leukocidin ED can deplete the frequencies of CD56^dim^ or CD56^hi^ NK cells^67^ while α-hemolysin^68^ and β-hemolysin^69^ can modulate the IFN-γ–producing capacity of NK cells. However, their effect on NKG2A expression in NK cells is unknown. Beyond this, it is possible that unique features of the immune response to *S. aureus* may promote this increased frequency of CD57^−^ NKG2A^+^ NK cells. IL-12 can upregulate NKG2A expression on NK cells,^75^^,^^76^ yet we found that plasma IL-12p70 levels were not significantly different in patients with *S. aureus* bacteremia compared to healthy controls. TGF-β is capable of increasing NKG2A expression on T cells,^85^ and TGF-β activation occurs in mouse models of *S. aureus* infection, principally through the actions of α-hemolysin.^86^ Thus, the increased frequency of CD57^−^ NKG2A^+^ NK cells could be mediated by TGF-β, although a lack of change in the levels of CD57^−^ NKG2A^+^ CD56^+^ T cells in patients with *S. aureus* bacteremia, compared to healthy controls, would not be consistent with this. Nevertheless, expanding our understanding of how *S. aureus* modulates NK cell activity is important to determine whether these effects are exclusive to *S. aureus* or are observed during infection with other bacterial pathogens.

In conjunction with observing an increase in the frequency of CD57^−^ NKG2A^+^ NK cells in patients with *S. aureus* bacteremia, we observed a significant reduction in NKp30^+^ Siglec-7^+^ CD56^dim^ NK cells in patients with *S. aureus* bacteremia. This could indicate that some element of NKp30 and Siglec-7 downregulation was occurring during *S. aureus* infection. While there is no existing evidence to prove this, it is known that TGF-β, which is activated during *S. aureus* infection *in vivo*,^86^ can downregulate NKp30 expression on NK cells.^87^ Furthermore, NK cells lacking Siglec-7 also have reduced NKp30 expression and display a diminished ability to degranulate and produce IFN-γ in response to PMA/ionomycin stimulation.^88^ However, our data suggest that *S. aureus* infection promotes a phenotypic shift toward NK cell subsets with greater IFN-γ–producing capacity, which contradicts this. Nevertheless, it highlights a need to perform more mechanistic investigations into why the number of NKp30^+^ Siglec-7^+^ NK cells are reduced in patients with *S. aureus* bacteremia. Regardless, this phenotypic change concurrently led to a significant increase in the percentage of NKp30^−^ Siglec-7^−^ CD56^dim^ NK cells compared to healthy controls. This NK cell subset includes adaptive NK cells, which are typically defined as highly differentiated CD57^+^ NKG2C^+^ populations.^46^^,^^47^^,^^77^^,^^78^ However, we did not observe a significant change in the percentage of CD57^+^ NKG2C^+^ cells within this NKp30^−^ Siglec-7^−^ subset in patients with *S. aureus* bacteremia. The CD57^+^ NKG2C^+^ adaptive NK cell phenotype was initially defined in HCMV-seropositive individuals and likely represents a feature of the host response to HCMV-encoded immune evasion.^46^^,^^47^^,^^77^^,^^78^ However, adaptive NK cells can also be generated in HCMV-seropositive individuals carrying a homozygous deletion in *NKG2C*. These *NKG2C*^−/−^ adaptive NK cells display similar functional and phenotypic attributes with CD57^+^ NKG2C^+^ equivalents, including high expression of leukocyte immunoglobulin-like receptor 1 (LIR1) and CD2,^77^^,^^78^^,^^89^^,^^90^ both of which are targeted by the HCMV-encoded MHC class I homologue, UL18, and the endoplasmic reticulum-resident glycoprotein, UL148, respectively.^91^^,^^92^ In our NKp30^−^ Siglec-7^−^ NK cell dataset, we did not observe any significant difference in CD57^+^ NKG2C^−^ cell subsets, nor even CD57^+^ NKG2A^+^ cells, in patients with *S. aureus* bacteremia. Instead, we saw a concurrent rise in the percentage of CD57^−^ NKG2A^+^ NK cells, akin to seen in all CD56^dim^ NK cells. This would further point toward a global effect of *S. aureus* infection in promoting a phenotypic shift toward CD57^−^ NKG2A^+^ NK cell populations, including in cells lacking NKp30 and Siglec-7. In line with these concepts, it is plausible that *S. aureus* may promote this NK cell phenotype by modulating expression of HLA-E, a nonclassical MHC class I molecule that presents self and viral peptides to NK cells, including polymorphic peptides derived from the HCMV-encoded UL40 protein.^93^ HLA-E binds to NKG2C or NKG2A in dimeric complexes with CD94 but possesses a greater affinity toward NKG2A,^94^^,^^95^ which delivers an inhibitory signal to NKG2A^+^ NK cells.^96^^,^^97^ Thus, *S. aureus*–induced changes in HLA-E expression could promote changes in the frequency of CD57^−^ NKG2A^+^ NK cell subsets. Furthermore, SAgs may also reduce the expression of HLA-E on different cell types including APCs releasing its inhibitory effect on NKG2A^98^ and leading to greater activation and expansion of CD57^−^ NKG2A^+^ NK cell populations. Further work is required to determine if this is a true feature of invasive *S. aureus* infection but also how, and if, severe bacterial infections may affect adaptive NK cell populations, including those lacking NKG2C.

The ability for SAgs from *S. aureus*, including TSST-1, to alter NK cell functionality has been reported before, notably in how they indirectly induce NK cells to produce high levels of IFN-γ through an IL-12–dependent mechanism.^65^^,^^66^ While our data agree with these observations, we discovered that SAgs, notably TSST-1, promoted the expansion of highly proliferative, IFN-γ–producing CD57^−^ NKG2A^+^ NK cells with reduced CD16 expression, but this process was not dependent on IL-12 even though SAgs promote IL-12 production from myeloid cells^66^^,^^80^^,^^81^ and IL-12 itself can upregulate NKG2A expression on NK cells.^75^^,^^76^ Consequently, the core mechanism involved requires elucidation. TSST-1 has been shown to increase the percentage of NKG2A^+^ T cells in time-course stimulation assays where PBMCs were depleted of CD94^+^ cells.^99^ Furthermore, while these effects were relatively modest, even after 8 to 10 days of stimulation, co-culture with IL-15 or TGF-β amplified this process significantly.^99^^,^^100^ This suggested that TSST-1 was effective at converting NKG2A^−^ T cells into NKG2A^+^ T cells in tandem with IL-15 or TGF-β. Furthermore, IL-12 supplementation had no effect on this process.^99^ As such, our data on SAg-mediated changes in NK cells may reflect NKG2C^−^ NKG2A^−^ NK cells converting into NKG2C^−^ NKG2A^+^ NK cell subsets, rather than specific proliferation events occurring solely in preexisting NKG2C^−^ NKG2A^+^ NK cells. However, it is worth noting that these T cell–based studies did not explore what factors were critical for TSST-1 being able to increase the percentage of NKG2A^+^ T cells in the absence of supplemented co-factors such as IL-15 or TGF-β. Recently, it was shown that SEB-induced changes in NKG2A expression on CD8^+^ T cells were impaired by CD40 blockade, which also prevented IL-12 production.^101^ However, it is still unknown if CD40 influences SAg-mediated changes in NKG2A expression on NK cells or the expansion of CD57^−^ NKG2A^+^ NK cells seen in our experiments. Given that SAgs have been reported to bind to CD40^26^, which is found on APCs, such binding could theoretically promote these phenotypic changes. Nonetheless, it will be important to dissect the critical factors that enable SAgs to increase the frequency of CD57^−^ NKG2A^+^ NK cells and compare such dependency patterns in T cells, where direct binding by SAgs to TCRs occurs. To this end, the notion that SAgs may directly bind NK cells^102^ has been refuted since human NK cells alone appear to be unresponsive to SAgs, only occurring when T cells and monocytes are co-cultured with them.^66^

Compared to what is known about NK cell–mediated immunity to viruses, our knowledge about how NK cells respond to bacterial infections is very limited and is complicated by the ambiguous nature of their precise role. Indeed, it appears that there are species-specific differences in the control and immunopathology of bacterial infections by NK cells. Our data on the NK cell response to *S. aureus* bacteremia supports the concept that NK cells can promote immune pathology during bacterial infection and that this is linked to the impact of NK cell–derived production of IFN-γ, and likely other cytokines, in driving excessive inflammation. However, more evidence is needed to examine the precise role of NK cells across different types of bacterial infection in humans. Indeed, our study had some limitations, notably in the number of patients with *S. aureus* or *E. coli* bacteremia that we enrolled. Thus, larger, sex-matched longitudinal studies are needed to expand this further and learn more about NK cell–mediated inflammation to systemic *S. aureus* infection. This is even more relevant given the increased mortality risk seen in female patients with *S. aureus* bacteremia,^103^^,^^104^ which ultimately means that a better understanding of sex-specific nuances in NK cell–mediated immunity is even more crucial. Furthermore, it will be important to compare findings with data collected from patients with bloodstream infections caused by other bacterial organisms. In this regard, bacteremia caused by *S. aureus*, especially MRSA, and other Gram-negative bacteria, such as *Klebsiella pneumoniae* and *Pseudomonas aeruginosa*, is associated with higher all-cause and infection-attributable mortality compared to *E. coli* bacteremia.^6^ Thus, it is plausible that specific changes in NK cell phenotype and function could be linked to infection severity and mortality. Consequently, it will be important to correlate the NK cell response to bacteremia with other measures of infection severity, such as Sequential Organ Failure Assessment score, to determine if NK cell–specific phenotypic changes are impacted by morbidity and infection-attributable or all-cause mortality.

In summary, we observed an increase in polyfunctional CD56^dim^ NK cells with greater cytokine-producing potential in patients with *S. aureus* bacteremia. These patients also exhibited increased frequencies of CD57^−^ NKG2A^+^ NK cell subsets, while SAgs from *S. aureus* promoted the expansion of IFN-γ–producing, CD57^−^ NKG2A^+^ NK cells through an IL-12–independent mechanism. As such, our data support the concept that immune pathogenesis during *S. aureus* infection may be linked to overproduction of proinflammatory cytokines and that alterations in NK cell–released IFN-γ could be a driver of immune pathogenesis during *S. aureus* infection. These mechanisms require further elucidation but imply that therapeutic approaches combining immune modulatory agents with antibiotics need to be explored.

## Supplementary Material

vkaf174_Supplementary_Data

## Data Availability

The data underlying this article are available in the article and in its online supplementary material along with being openly available in the Cardiff University Research Data Repository at https://doi.org/10.17035/cardiff.29366246.
