# Supplementary material for: NKG2A-mediated immune modulation of natural killer cells by Staphylococcus aureus
Source: J Immunol. 2025 Aug 7;214(12):3332–44. doi: 10.1093/jimmun/vkaf174 (PMC12726069; doi:10.1093/jimmun/vkaf174)
Supplement: vkaf174_Supplementary_Data [file vkaf174_supplementary_data.zip › Davies et al supplementary material FINAL - Jun 2025.pdf]

SUPPLEMENTARY MATERIAL

NKG2A-mediated immune modulation of natural killer cells by *Staphylococcus aureus*

Kate Davies*, Al-Motaz Rizek*, Sarah Edkins*, [†], Simon Kollnberger*, [†], Eddie C. Y. Wang*, [†], Matthias Eberl*, [†], Jonathan Underwood*, [†, ‡] and James E. McLaren*, [†, §]

*Division of Infection and Immunity, Cardiff University School of Medicine, Cardiff, UK

[†]Systems Immunity University Research Institute, Cardiff University School of Medicine, Cardiff, UK

[‡]Department of Infectious Diseases, Cardiff and Vale University Health Board, Heath Park, Cardiff, CF14 4XW, UK

[§]Corresponding author: Dr James E McLaren.

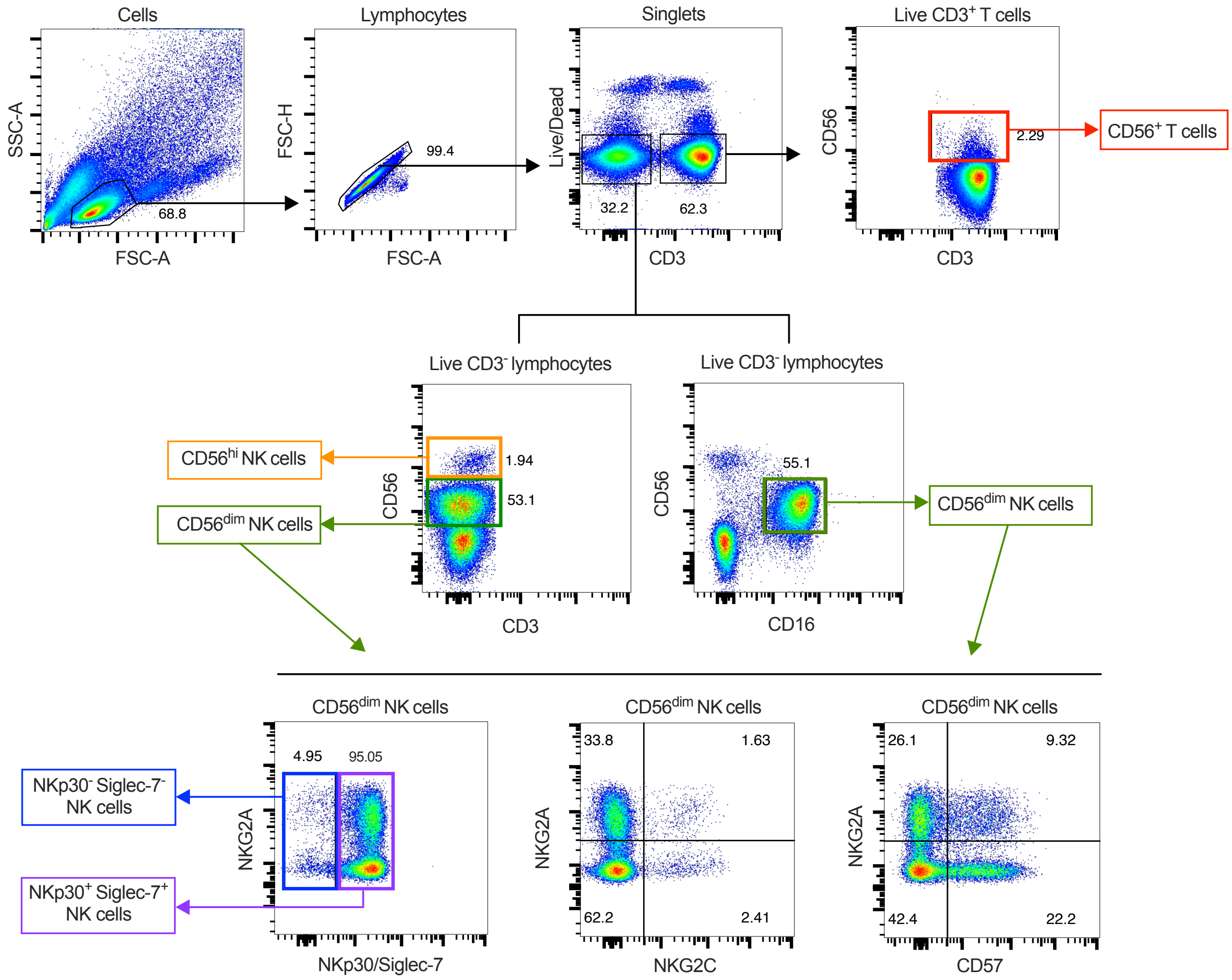

Figure S1 – Gating strategy for identifying NK cell and CD56^{+} T cell populations in human PBMCs

Successive flow cytometric panels depicting the gating strategy used to identify viable CD56^{+} T cells, CD56^{dim} NK cells or CD56^{hi} NK cells in human PBMCs from a representative donor. Additional flow cytometric panels depict the gating strategy for identifying NKp30^{-} Siglec-7^{-} or NKp30^{+} Siglec-7^{+} CD56^{dim} NK cells and NK cell subsets segregated based on NKG2A vs NKG2C expression or NKG2A vs CD57 expression.

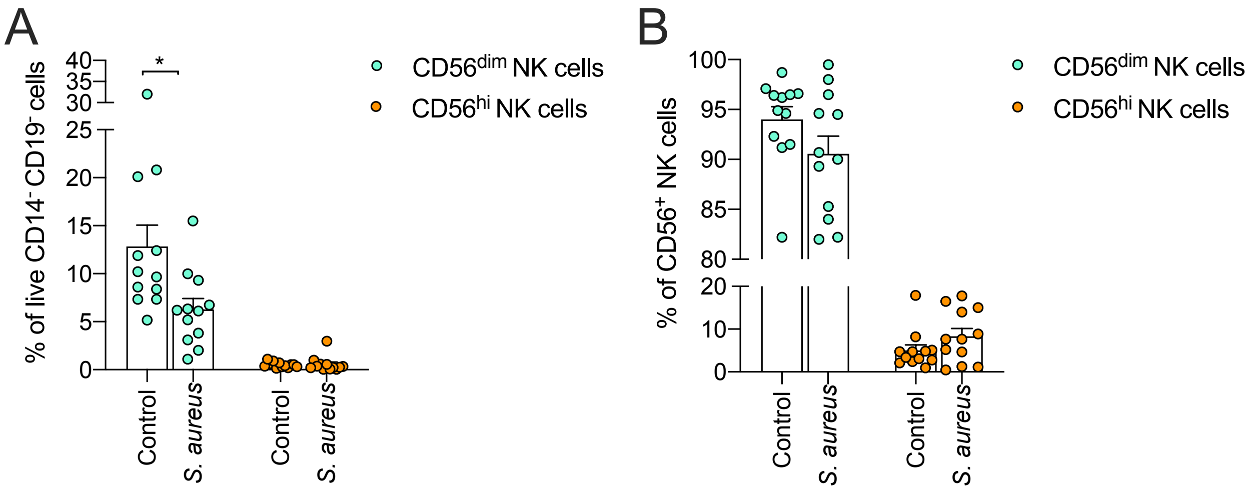

Figure S2 – NK cell frequencies in healthy controls and patients hospitalized with *S. aureus* bacteraemia

Frequency of $CD56^{dim}$ (green filled circles) or $CD56^{hi}$ (orange filled circles) NK cells as a percentage of viable, $CD14^{-}$ $CD19^{-}$ cells (A) or $CD56^{+}$ $CD3^{-}$ NK cells (B) from human PBMCs collected from healthy controls and patients hospitalized with *S. aureus* or *E. coli* bacteraemia. Each dot represents one control or patient. Data are shown as mean ± SEM; $^{*}p < 0.05$, unpaired t-test.

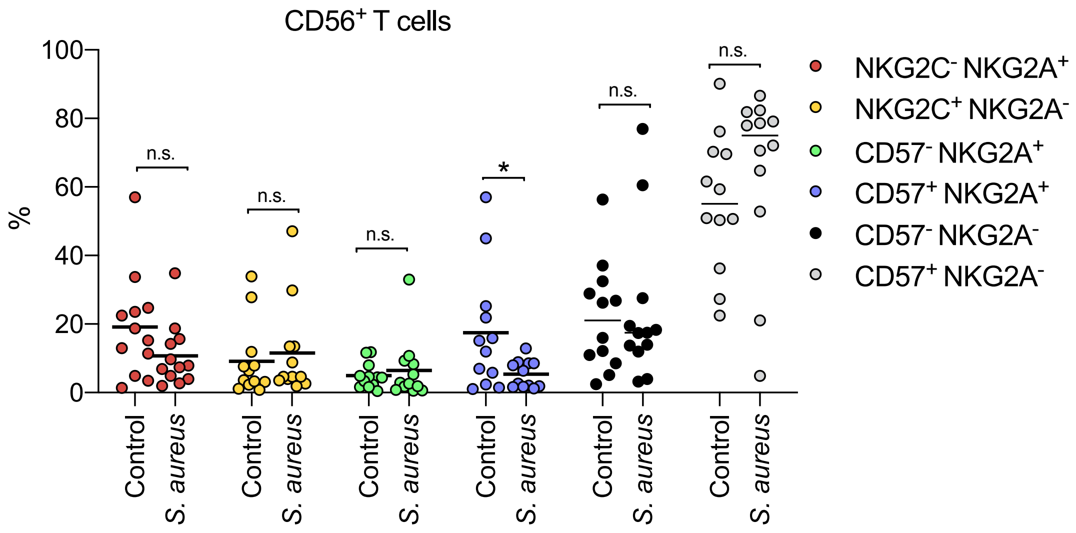

Figure S3 – Phenotypic analyses of CD56$^+$ T cells in healthy controls and patients hospitalized with *S. aureus* bacteraemia

Scatter dot plot showing the frequency of NKG2C$^-$ NKG2A$^+$ (red filled circles), NKG2C$^+$ NKG2A$^-$ (yellow filled circles), CD57$^-$ NKG2A$^+$ (green filled circles), CD57$^+$ NKG2A$^+$ (light blue filled circles), CD57$^-$ NKG2A$^-$ (black filled circles) or CD57$^+$ NKG2A$^-$ (grey filled circles) subsets among CD56$^+$ T cells from human PBMCs collected from healthy controls and patients hospitalized with *S. aureus* bacteraemia. Each dot represents one control or patient. Line indicates mean value. *$p<0.05$; unpaired t-test. n.s. = not significant.

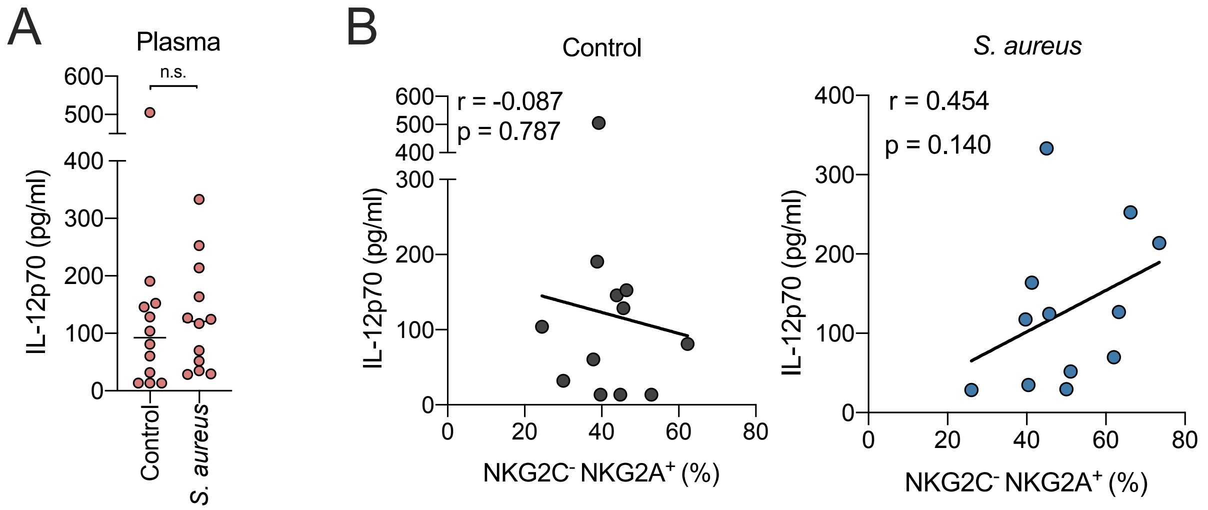

Figure S4 – Plasma IL-12p70 levels are not reduced in patients with *S. aureus* bacteraemia

(A) IL-12p70 protein levels (pg/ml) in plasma collected from healthy controls and patients hospitalized with *S. aureus* bacteraemia. Each dot represents one control or patient. Line indicates mean value. n.s. = not significant (B) Spearman rank correlations between plasma IL-12p70 levels and the percentage of NKG2C^{-} NKG2A^{+} subsets among CD56^{dim} NK cells from human PBMCs collected healthy controls (black filled circles) or patients hospitalized with *S. aureus* (blue filled circles) bacteraemia. Each dot represents one control or patient. r and p values are depicted on each graph.

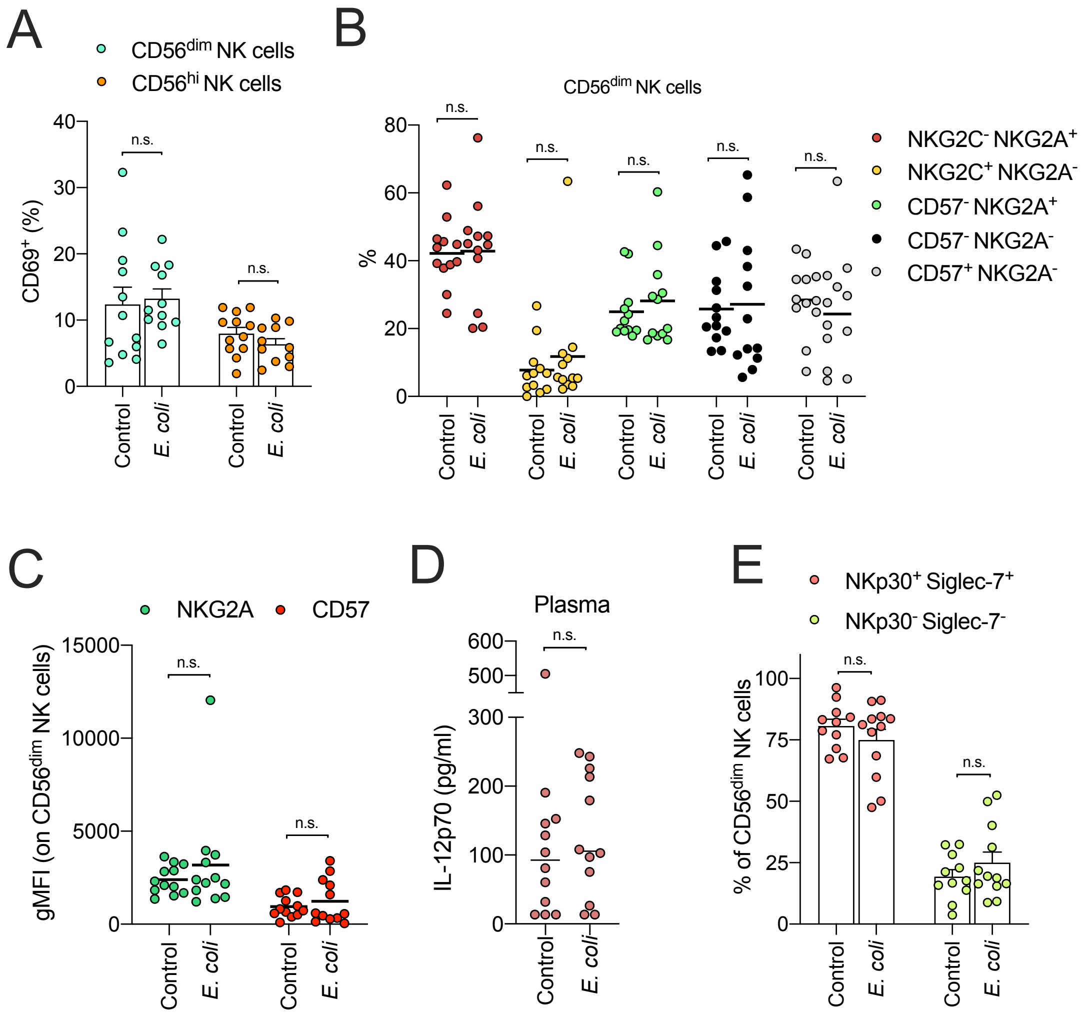

Figure S5 – CD57⁻ NKG2A⁺ or NKp30⁻ Siglec-7⁻ NK cells do not increase in frequency *in vivo* during *E. coli* bacteraemia in humans

(A) Frequency of CD69⁺ cells among CD56^{dim} (green filled circles) or CD56^{hi} (orange filled circles) NK cells from human PBMCs collected from healthy controls and patients hospitalized with *E. coli* bacteraemia. Each dot represents one control or patient. Data are shown as mean ± SEM. n.s. = not significant. (B) Scatter dot plot showing the frequency of NKG2C⁻ NKG2A⁺ (red filled circles), NKG2C⁺ NKG2A⁻ (yellow filled circles), CD57⁻ NKG2A⁺ (green filled circles), CD57⁻ NKG2A⁻ (black filled circles) or CD57⁺ NKG2A⁻ (grey filled circles) subsets among CD56^{dim} NK cells from human PBMCs collected from healthy controls and patients

hospitalized with *E. coli* bacteraemia. Each dot represents one control or patient. Line indicates mean value. n.s. = not significant. (C) Scatter dot plots depicting geometric mean fluorescence intensity (gMFI) of NKG2A (green filled circles) or CD57 (red filled circles) expression on CD56^{dim} NK cells from human PBMCs collected from healthy controls and patients hospitalized with *E. coli* bacteraemia. Each dot represents one donor. Line indicates mean value. n.s. = not significant. (D) IL-12p70 protein levels (pg/ml) in plasma collected from healthy controls and patients hospitalized with *E. coli* bacteraemia. Each dot represents one control or patient. Line indicates mean value. n.s. = not significant. (E) (A) Frequency of NKp30^{+} Siglec-7^{+} (mid pink filled circles) or NKp30^{-} Siglec-7^{-} (light green filled circles) among CD56^{dim} NK cells from human PBMCs collected from healthy controls and patients hospitalized with *E. coli* bacteraemia. Each dot represents one control or patient. Data are shown as mean ± SEM. n.s. = not significant.

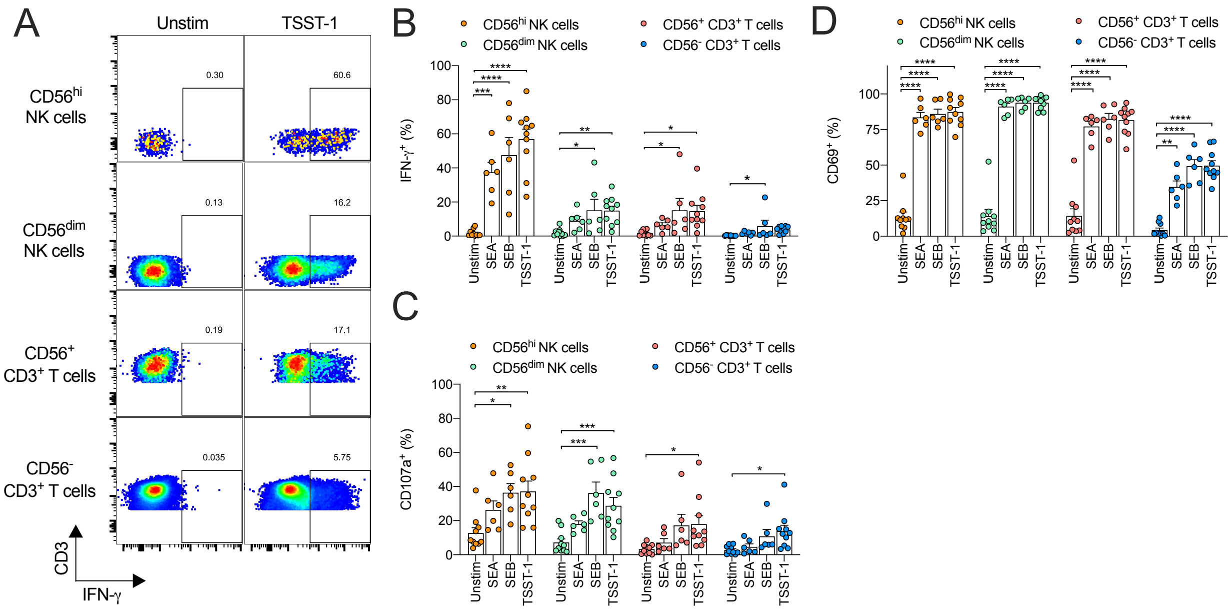

Figure S6 – CD56 positivity in NK cells and T cells dictate hyperresponsiveness to SAgs

(A) Representative flow cytometry plots depicting expression of CD3 vs IFN-γ on CD56^{hi} NK cells, CD56^{dim} NK cells, CD56^{+} CD3^{+} T cells and CD56^{-} CD3^{+} T cells from human PBMCs collected from healthy controls that were cultured in medium alone (Unstim) or stimulated with TSST-1 for 24h. (B-D) Frequency of IFN-γ^{+} (B), CD107a^{+} (C) or CD69^{+} (D) cells among CD56^{hi} NK cells (orange filled circles), CD56^{dim} NK cells (green filled circles), CD56^{+} CD3^{+} T cells (pink filled circles) and CD56^{-} CD3^{+} T cells (blue filled circles) from human PBMCs collected from healthy controls that were cultured in medium alone (Unstim) or stimulated with SEA, SEB or TSST-1 for 24h. Each dot represents one donor. Data are shown as mean ± SEM. *$p < 0.05$, **$p<0.01$, ***$p < 0.001$, ****$p<0.0001$; One-way ANOVA with Tukey's post-hoc test.

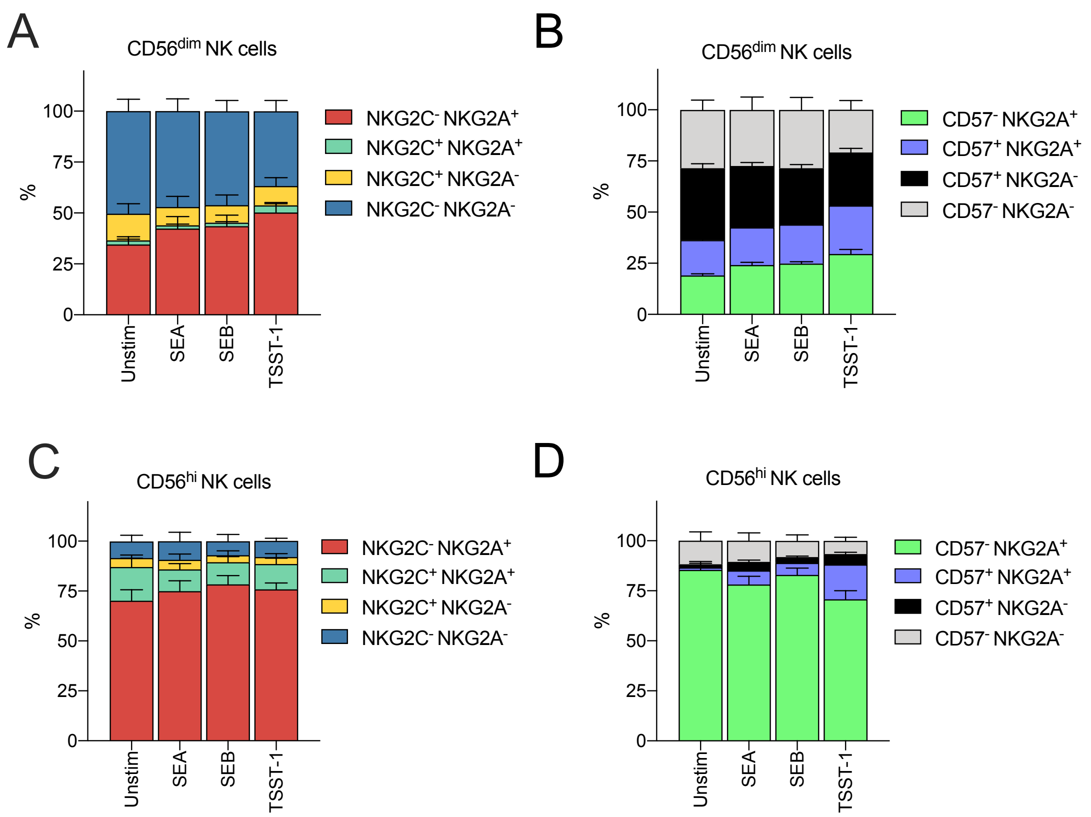

Figure S7 – Phenotypic analyses of CD56^{dim} and CD56^{hi} NK cells following SAg stimulation

Stacked bar charts showing the frequency of NKG2C^{-} NKG2A^{+}, NKG2C^{+} NKG2A^{+}, NKG2C^{+} NKG2A^{-} and NKG2C^{-} NKG2A^{-} (A, C) or CD57^{-} NKG2A^{+}, CD57^{+} NKG2A^{+}, CD57^{+} NKG2A^{-}, CD57^{-} NKG2A^{-} (B, D) subsets among CD56^{dim} NK cells (A, B) or CD56^{hi} NK cells (C, D) from human PBMCs collected from healthy controls that were cultured in medium alone (Unstim) or stimulated with SEA (*n* = 6), SEB (*n* = 6) or TSST-1 for 24h (*n* = 10).

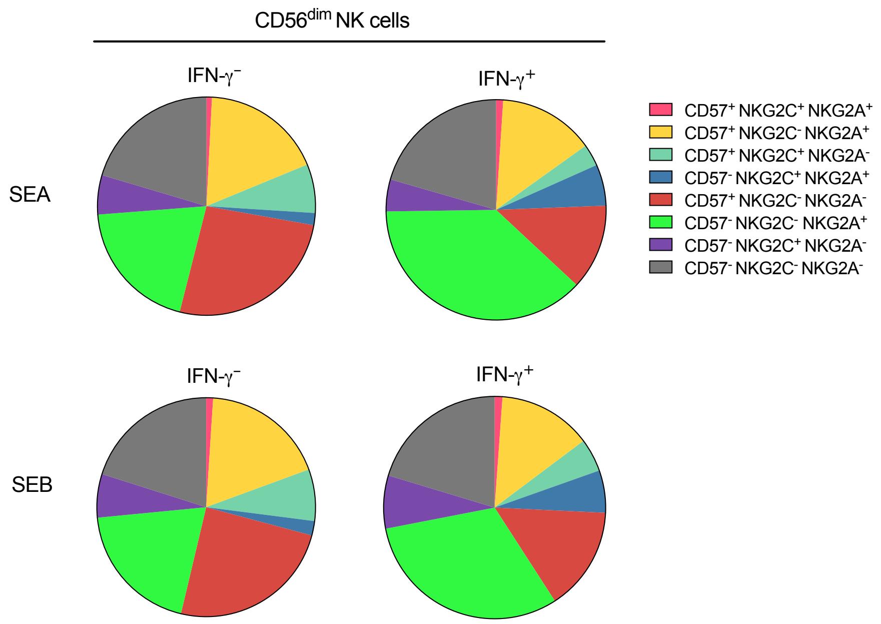

Figure S8 – IFN-γ production in response to SAg stimulation is mostly confined to $CD57^{-}$ $NKG2C^{-}$ $NKG2A^{+}$ NK cell subsets

Pie charts depicting the proportion of IFN-γ^{-} or IFN-γ^{+} $CD56^{dim}$ NK cells that are made up of subsets that express combinations of CD57, NKG2A and/or NKG2C from human PBMCs that were stimulated with SEA or SEB for 24h. Data is concatenated from *n* = 6 individual donors

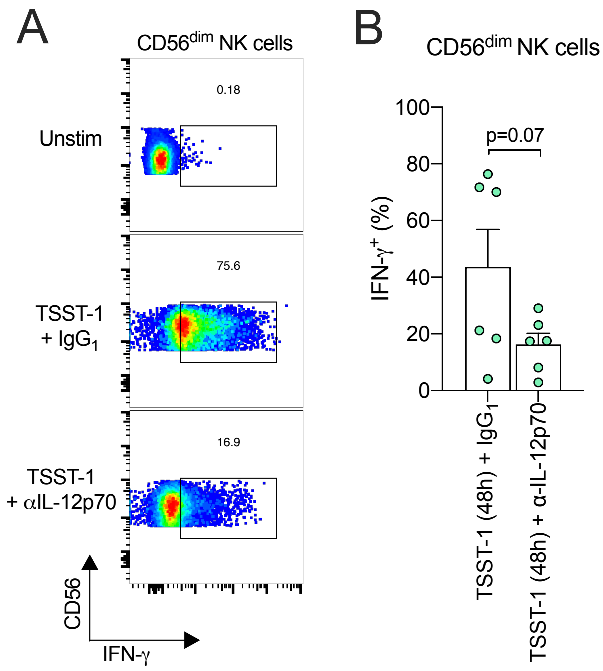

Figure S9 – The percentage of IFN-γ^+ CD56^{dim} NK cells elicited after TSST-1 stimulation following IL-12 blockade

(A) Representative flow cytometry plots depicting expression of CD56 vs IFN-γ on CD56^{dim} NK cells from human PBMCs collected from healthy controls that were cultured in medium alone (Unstim) or pretreated with anti-human IL-12p70 or an isotype control antibody (IgG_1) before being stimulated with TSST-1 for 48h. (B) Frequency of IFN-γ^+ cells among CD56^{dim} NK cells (green filled circles) from human PBMCs collected from healthy controls that were pretreated with anti-human IL-12p70 or an isotype control antibody (IgG_1) before being stimulated with TSST-1 for 48h. Each dot represents one donor. Data are shown as mean ± SEM.
